# Functional annotation of rhizospheric phageome of the wild plant species *Moringa oleifera*

**DOI:** 10.3389/fmicb.2023.1166148

**Published:** 2023-05-16

**Authors:** Ruba A. Ashy, Rewaa S. Jalal, Hana S. Sonbol, Mashael D. Alqahtani, Fatmah O. Sefrji, Sahar A. Alshareef, Fatimah M. Alshehrei, Haneen W. Abuauf, Lina Baz, Manal A. Tashkandi, Israa J. Hakeem, Mohammed Y. Refai, Aala A. Abulfaraj

**Affiliations:** ^1^Department of Biology, College of Science, University of Jeddah, Jeddah, Saudi Arabia; ^2^Department of Biology, College of Sciences, Princess Nourah bint Abdulrahman University, Riyadh, Saudi Arabia; ^3^Department of Biology, College of Science, Taibah University, Al-Madinah Al-Munawwarah, Saudi Arabia; ^4^Department of Biology, College of Science and Arts at Khulis, University of Jeddah, Jeddah, Saudi Arabia; ^5^Department of Biology, Jumum College University, Umm Al-Qura University, Makkah, Saudi Arabia; ^6^Department of Biology, Faculty of Applied Science, Umm Al-Qura University, Makkah, Saudi Arabia; ^7^Department of Biochemistry, Faculty of Science, King AbdulAziz University, Jeddah, Saudi Arabia; ^8^Department of Biochemistry, College of Science, University of Jeddah, Jeddah, Saudi Arabia; ^9^Biological Sciences Department, College of Science & Arts, King AbdulAziz University, Rabigh, Saudi Arabia

**Keywords:** CAZyme, KEGG, ARG, HGT, cell membrane, antibiotics

## Abstract

**Introduction:**

The study aims to describe phageome of soil rhizosphere of *M.oleifera* in terms of the genes encoding CAZymes and other KEGG enzymes.

**Methods:**

Genes of the rhizospheric virome of the wild plant species *Moringa oleifera* were investigated for their ability to encode useful CAZymes and other KEGG (Kyoto Encyclopedia of Genes and Genomes) enzymes and to resist antibiotic resistance genes (ARGs) in the soil.

**Results:**

Abundance of these genes was higher in the rhizospheric microbiome than in the bulk soil. Detected viral families include the plant viral family Potyviridae as well as the tailed bacteriophages of class Caudoviricetes that are mainly associated with bacterial genera *Pseudomonas, Streptomyces* and *Mycobacterium*. Viral CAZymes in this soil mainly belong to glycoside hydrolase (GH) families GH43 and GH23. Some of these CAZymes participate in a KEGG pathway with actions included debranching and degradation of hemicellulose. Other actions include biosynthesizing biopolymer of the bacterial cell wall and the layered cell wall structure of peptidoglycan. Other CAZymes promote plant physiological activities such as cell-cell recognition, embryogenesis and programmed cell death (PCD). Enzymes of other pathways help reduce the level of soil H_2_O_2_ and participate in the biosynthesis of glycine, malate, isoprenoids, as well as isoprene that protects plant from heat stress. Other enzymes act in promoting both the permeability of bacterial peroxisome membrane and carbon fixation in plants. Some enzymes participate in a balanced supply of dNTPs, successful DNA replication and mismatch repair during bacterial cell division. They also catalyze the release of signal peptides from bacterial membrane prolipoproteins. Phages with the most highly abundant antibiotic resistance genes (ARGs) transduce species of bacterial genera *Pseudomonas, Streptomyces*, and *Mycobacterium*. Abundant mechanisms of antibiotic resistance in the rhizosphere include “antibiotic efflux pump” for ARGs *soxR, OleC*, and *MuxB*, “antibiotic target alteration” for *parY mutant*, and “antibiotic inactivation” for *arr-1*.

**Discussion:**

These ARGs can act synergistically to inhibit several antibiotics including tetracycline, penam, cephalosporin, rifamycins, aminocoumarin, and oleandomycin. The study highlighted the issue of horizontal transfer of ARGs to clinical isolates and human gut microbiome.

## 1. Introduction

*Moringa oleifera* is an edible wild plant that can withstand adverse environmental conditions. This plant is native to several countries, including Brazil, Egypt, India, Pakistan, Thailand, and Saudi Arabia (Al-Eisawi and Al-Ruzayza, 2015; Gupta and Ahmed, 2020). *M. oleifera* is very useful at medicinal and nutritional levels as it is a good source of proteins and vitamins, in addition to being rich in beta-carotene and phenolics. It also acts as cardiac and circulatory stimulants and lowers blood pressure and cholesterol levels (Fahey, 2005). Some parts of this wild have several pharmaceutical properties, such as anti-cancer, anti-inflammatory, anti-oxidant, anti-diabetic, anti-bacterial, and anti-fungal (Koul and Chase, 2015; Kumar et al., 2016; Saini et al., 2016; Gupta and Ahmed, 2020; Milla et al., 2021). It also has agricultural applications including water purification and is suitable for human consumption, and some parts of the plant can be used as animal fodder, fertilizer, and livestock forage (Rockwood et al., 2013).

Gene cataloging via metagenomic whole-genome sequencing approach allows the detection of genes of rhizospheric soil microbiomes such as bacteria, archaea, eukaryotic microorganisms, and viruses (Vorholt et al., 2017; Odelade and Babalola, 2019). Functional annotation of metagenomes allows studying antibiotic resistance genes (ARGs) and those involved in Carbohydrate-Active enZyme (CAZyme) enrichment and cross-talking Kyoto Encyclopedia of Genes and Genomes (KEGG) pathways. The latter three gene categories participate in the interaction between microbiomes and intact plant roots, which results in shaping microbiome signatures and in the assemblage of beneficial microbial communities, on the one hand, and in promoting plant growth and development, on the other hand (Raes et al., 2007).

Differential abundance of microbes in the soil rhizosphere is mainly due to their differential response to varying chemical compositions of the plant root exudates that seem to affect microbial growth dynamics, biomass, diversity, community assembly, and metabolic potential (Pett-Ridge et al., 2021). It was recently reported that microbiomes of the microbial communities in plant rhizosphere are highly abundant in genes encoding CAZymes that act in building/degrading soil carbohydrates (Lombard et al., 2014; Levy et al., 2018). CAZymes with known functions were assigned to CAZy classes (level 1) and families (level 2), received enzyme classification (EC) codes (level EC), and then were deposited in CAZy (http://www.cazy.org/) and CAZypedia (https://www.cazypedia.org/) databases. The high potential of the rhizosphere genome translates into a high rate of complex carbohydrate build-up/degradation, which differs from one environmental niche to the other (Nuccio et al., 2020).

Metagenomic analysis via the KEGG database represents another dimension of the functional annotation of genes and proteins in soil microbiomes and the detection of cross-talking pathways as a response to plant–microbe interactions (Kanehisa et al., 2016). In the year 2015, KEGG authorities added categories of viruses and plasmids to the KEGG database as mobile genetic elements (MGEs) and as key players of the metagenome signature and function. Thus, gene catalog profiling can result in the detection of genes that likely exist in such MGEs to be horizontally transduced from one microbe to another genetically related microbe via specific patterns of horizontal gene transfer (HGT). This speculation raises the concern that ARGs possibly exist in mobile genetic elements (MGEs) of rhizospheric bacteria, which might pass through an edible plant, such as *M. oleifera*, to be eventually transmitted to the human gut or skin microbiome and transform/transduce pathogenic bacteria, mostly of similar species (Chen et al., 2019). However, there is not enough information regarding the type and severity of new versatile ARGs that possibly exist in the wild plant rhizosphere (Obermeier et al., 2021). Recent reports showed the possible influence of MGEs in HGT when studying metagenomics of freshwater and wastewater and demonstrated the occurrence of a large number of functional antibiotic resistance genes (ARGs) in their phageomes (Moon et al., 2020; Wang et al., 2021). The most dominant phage families known to harbor ARGs belong to tailed bacteriophages of the class Caudoviricetes, while the most abundant ARGs in this class are those for tetracycline and rifampicin resistance. Interestingly, phages likely incline to select specific ARGs, especially those encoding ribosomal protection proteins and RNA polymerases, e.g., subtypes *lsaE, tet44, tetM, tetP, macB, MdlB*, and *rpoB* (Wang et al., 2021).

The term horizontal gene transfer (HGT) refers to the transfer of genetic information mostly among genetically related organisms. When this action includes ARGs, it serves in fueling the evolution of pathogenic organisms (Burmeister, 2015). In total, three mechanisms of bacterial HGT are known: via the uptake of DNA from the environment, conjugation via the direct transfer of DNA from a bacterium to the conjugated ones, and transduction where transferred DNA is packed in bacteriophages. The HGT of ARGs serves effectively in the evolution of bacterial cells due to the plasticity of bacterial genomes and occurrence of selection pressure, which promote the competence of bacterial cells with potential for adaptability (Prudhomme et al., 2006; Peterson and Kaur, 2018). Ecological settings that mediate the occurrence of HGT include sewage, hospital effluents, and aquaculture, where the density of bacteria and mobile genetic elements (MGEs) is high (Resch et al., 2005; Modi et al., 2013; Stanczak-Mrozek et al., 2015; Von Wintersdorff et al., 2016).

In the present study, we have studied the phageome of the soil rhizosphere of *M. oleifera* in terms of the genes encoding CAZymes and other KEGG enzymes. We also studied the possibility of soil phageome to transduce ARGs from one bacterial taxon to a genetically related taxon, a process called horizontal gene transfer (HGT), and investigated its possibly accompanied risks to humans and the environment.

## 2. Materials and methods

### 2.1. Soil sample collection, DNA isolation, and sequencing

Soil samples at coordinates 21°1′17.8^′′^N 39°31′26.4^′′^E near Jeddah, Saudi Arabia (Al-Eisawi and Al-Ruzayza, 2015), were collected from the rhizosphere region of three single-grown, similar-in-size *M. oleifera* trees in addition to three samples of the surrounding bulk soil (≥10 m apart from the trees), as previously described (Hurt et al., 2001). Then, DNA was isolated, and an amount of 30 μl of each DNA sample (10 ng/μl) was shipped to Novogene Co. (Singapore) for whole-metagenomic sequencing using the Illumina HiSeq 2500 platform. Then, raw data were deposited in the European Nucleotide Archive (ENA) (https://www.ebi.ac.uk/ena/browser/), with accession nos. ERR10100770-74 and ERR10100781.

### 2.2. Bioinformatics and functional annotation of viruses

A library was prepared using the NEBNext^®^ UltraTM DNA Library Prep kit, while steps of dataset assembly and layers of quality control were performed as described (Karlsson et al., 2012; Mende et al., 2012; Oh et al., 2014). Library preparation entails DNA fragmentation, end repair 5‘ phosphorylation, and dA-tailing, followed by adaptor ligation, U excision, PCR enrichment, and DNA clean-up. Clean reads were assembled by Novogene Co., while mix less abundant unassembled reads of all samples to be resembles and recovered NOVO_MIX scaffolds of which scaftigs were generated as described (Mende et al., 2012; Nielsen et al., 2014). Assembled ORFs/NOVO_MIX queries were mapped against Soap 2.21 (default version), and genes were predicted and dereplicated by MetaGeneMark (Nielsen et al., 2014) and Cluster Database at High Identity with Tolerance (CD-HIT) (Li and Godzik, 2006; Fu et al., 2012), respectively. Non-redundant gene catalogs (nrGCs) were generated by greedy pairwise comparison (Li et al., 2014) and annotated by MEGAN (Huson et al., 2007). Then, functional abundance was generated using Diamond (Buchfink et al., 2015), and deduced amino acid sequences were mapped to the eggNOG database (version 4.0) (Huson et al., 2011, 2016; Powell et al., 2014; Buchfink et al., 2015; Huerta-Cepas et al., 2017) against the CAZy database (version 2014.11.25) (Lombard et al., 2014) to detect and assign CAZymes to their classes/families and draw pathways using the KEGG database (https://www.genome.jp/kegg/pathway.html). In terms of other KEGG enzymes, the KEGG Orthology (KO) database was used to detect molecular functions, while the KEGG PATHWAY database was used to map pathways at three KEGG levels (1, 2, and 3), and the KEGG ENZYME (EC or enzyme commission) database was used to functionally annotate ORFs/NOVO_MIX queries (Karlsson et al., 2012, 2013; Li et al., 2014). ARGs and gene queries were further mapped against the Comprehensive Antibiotic Resistance Database (CARD, https://card.mcmaster.ca/ontology/) (e-value ≤1^e − 5^) (Martínez et al., 2015), and gene abundance was estimated (Yang et al., 2013; Forsberg et al., 2014). ARGs were manually categorized into antimicrobial resistance (AMR) families and their resistance mechanisms, as described (Liu and Pop, 2009). Detected phage ARGs were searched for their bacterial hosts in the soil microbiome of *M. oleifera* in order to elaborate on the effects of bacterial transduction and subsequent biological events.

## 3. Results

**Figure 1:**
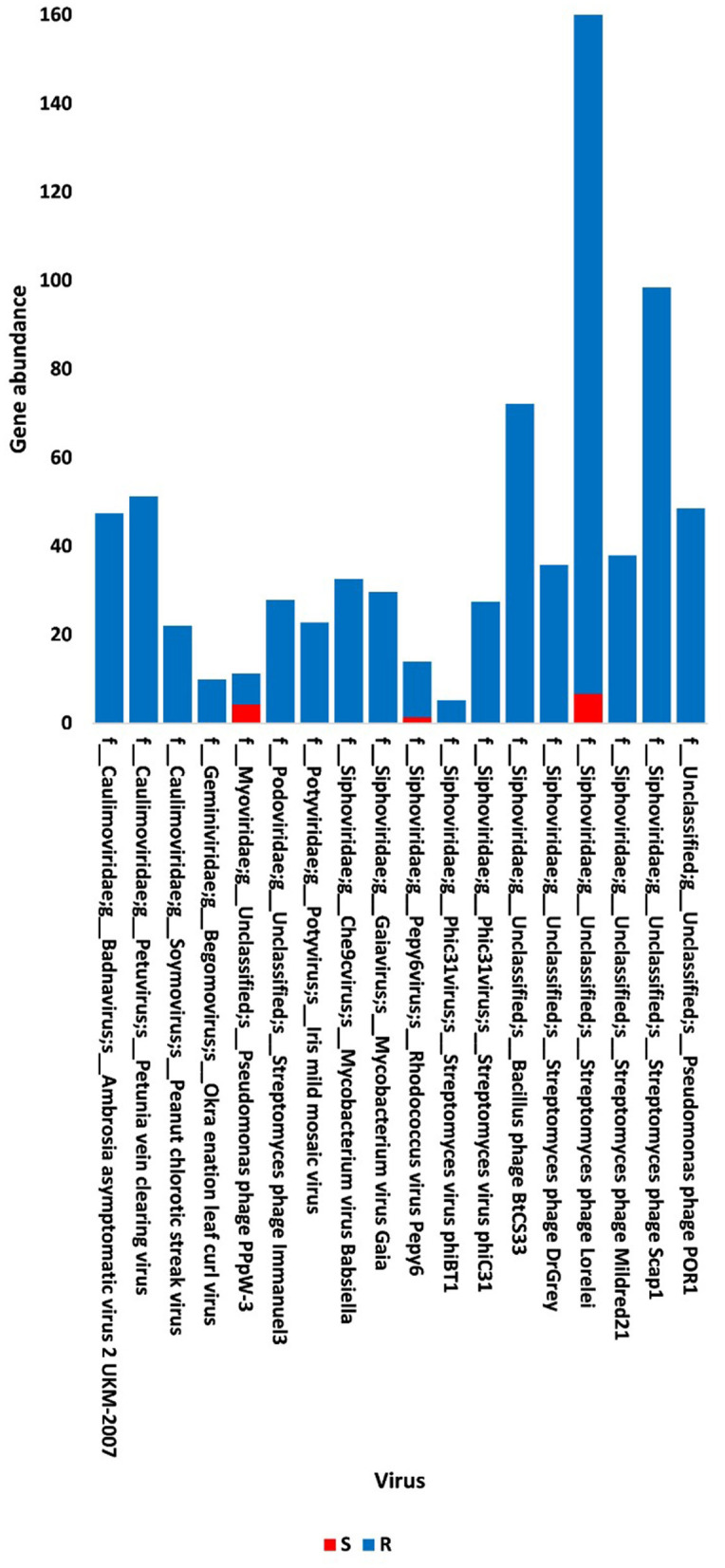
Abundance of viral non-redundant genes in soil, e.g., bulk (S) and rhizosphere (R), microbiomes of *Moringa oleifera*. For more taxonomic details, see Supplementary Table S2.

**Figure 2:**
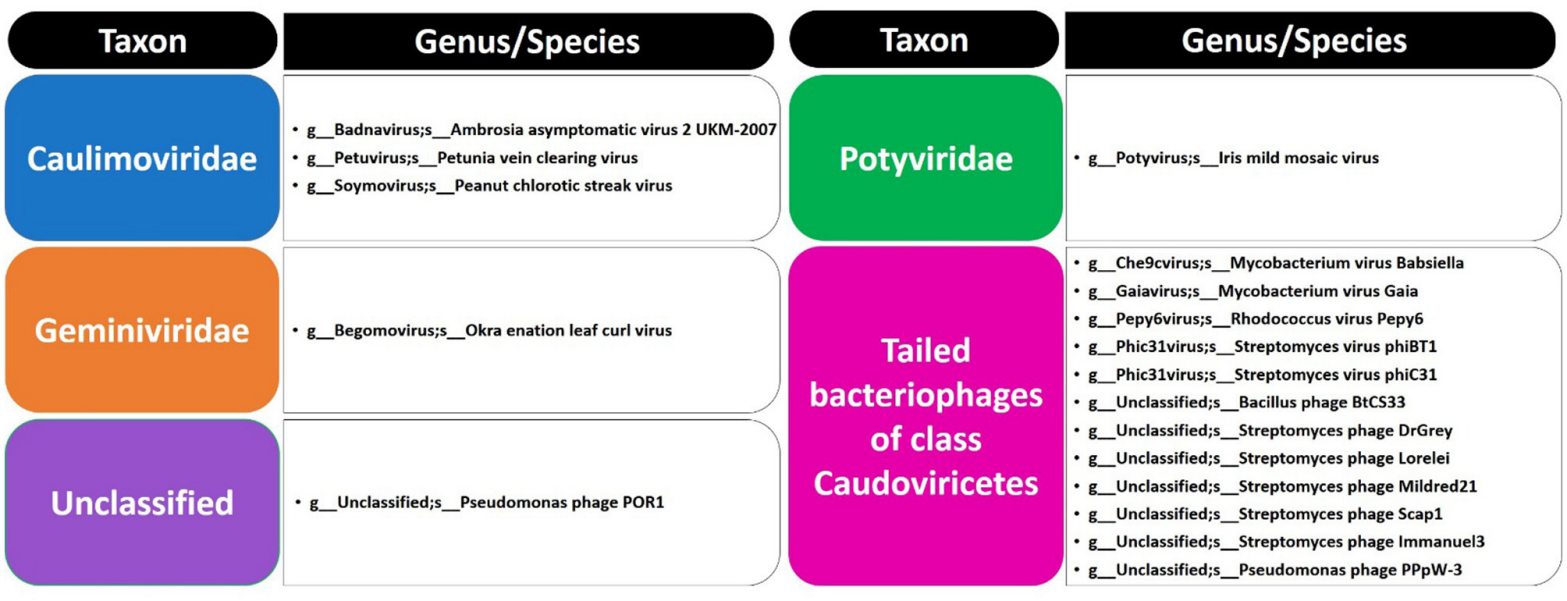
List of phages at family/genus/species levels in soil microbiomes of *Moringa oleifera*.

**Figure 3:**
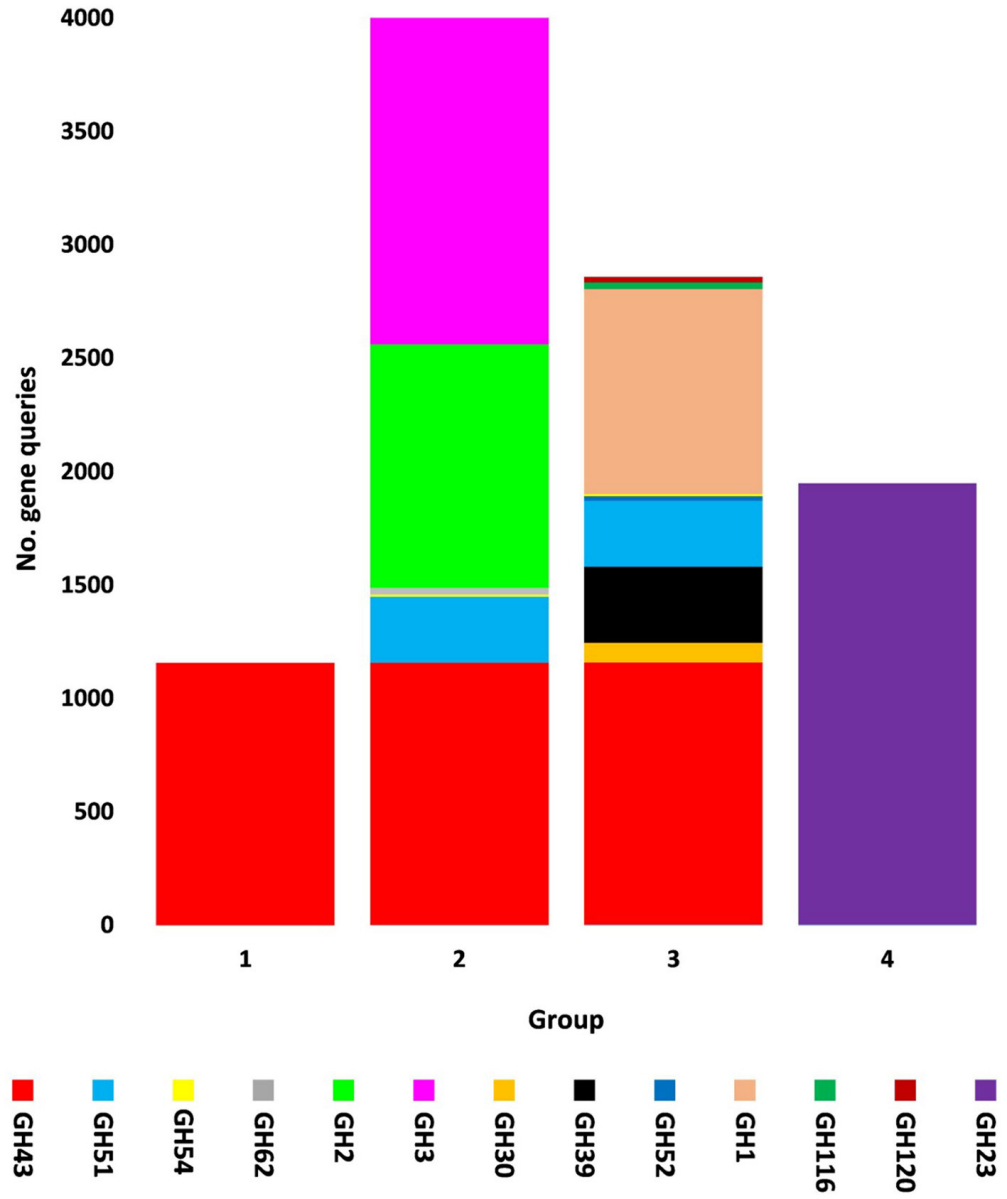
Query number of viral genes encoding CAZymes of CAZy glycoside hydrolase (GH) class along with their groups in soil microbiomes of *Moringa oleifera* across soil types. Group 1 = GH43, Group 2 = GH43/GH51/GH54/GH62/GH2/GH3, Group 3 = GH43/GH30/GH39/GH51/GH52/ GH54/GH1/GH116/GH120, and Group 4 = GH23. For more details, see Supplementary Table S7.

**Figure 4:**
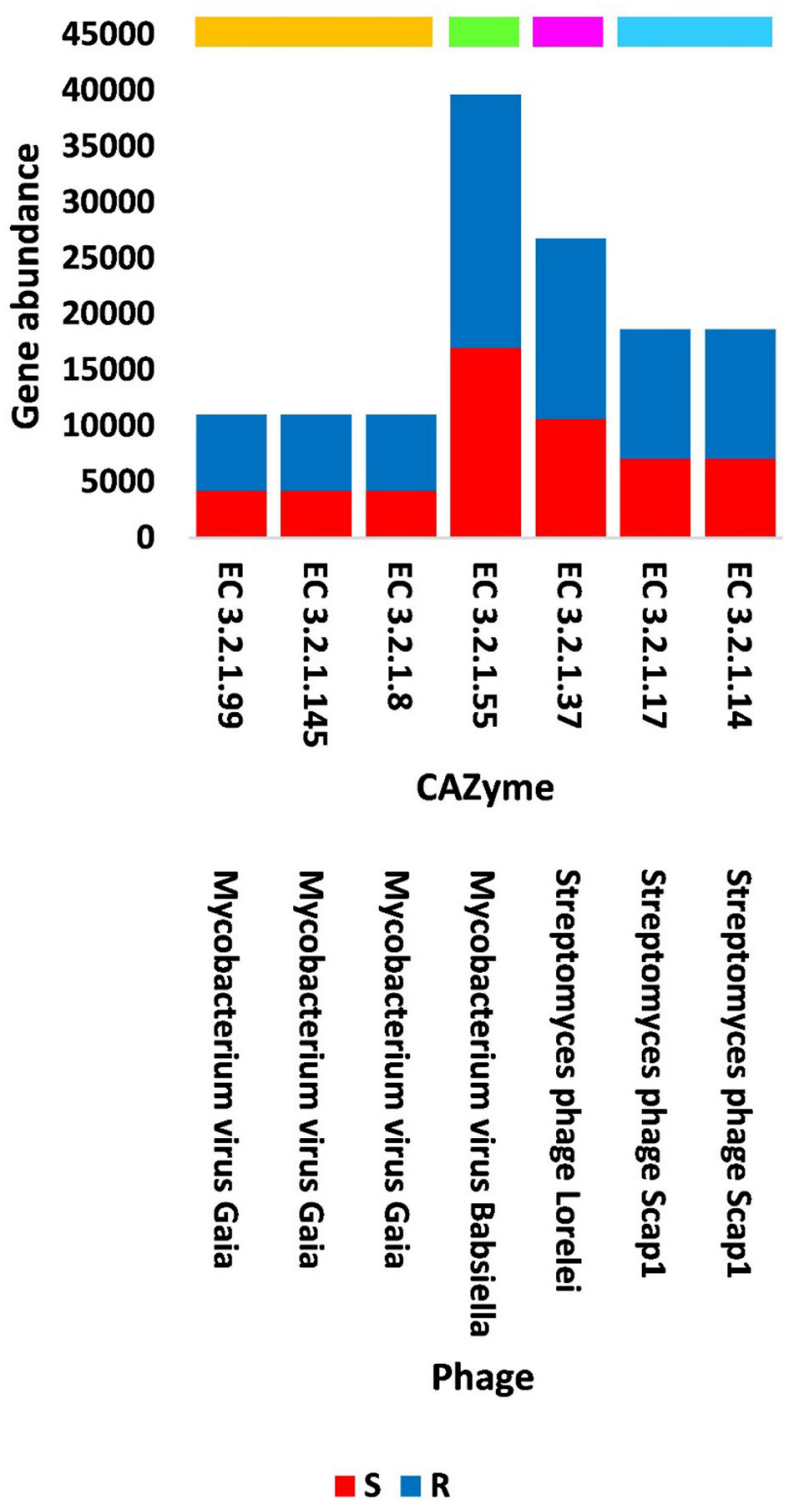
Abundance of viral genes encoding GH CAZyme groups in soil, e.g., bulk (S) and rhizosphere (R), microbiomes of *Moringa oleifera*. Orange box = CAZyme group 1, green box = CAZyme group 2, pink box = CAZyme group 3, blue box = CAZyme group 4. EC 3.2.1.99 = arabinanase, EC 3.2.1.145 = galactan 1,3-beta-galactosidase, EC 3.2.1.8 = endo-beta-1,4-xylanase, EC 3.2.1.55 = alpha-L- arabinofuranosidase, EC 3.2.1.37 = beta-xylosidase, EC 3.2.1.17 = lysozyme type G, and EC 3.2.1.14 = chitinase. For more details, see Supplementary Table S9.

The present study focused on the detection of viral genes that contribute to the functions assigned to the rhizospheric microbiome of *M. oleifera* in three categories, namely, genes encoding CAZymes, genes encoding other KEGG enzymes, and antibiotic resistance genes (ARGs). First, the abundance of viral genes was detected and proven to be higher in plant rhizosphere soil than in bulk soil (Figure 1, Supplementary Tables S1, S2). The number of overall detected viruses was 18, mostly belonging to tailed bacteriophages of class Caudoviricetes (12) followed by Caulimoviridae (3), Geminiviridae (1), Potyviridae (1), and one unclassified family (Figure 2, Supplementary Table S2). Of these, 13 viruses were bacteriophages of bacterial genera *Pseudomonas* (2), *Streptomyces* (7), *Mycobacterium* (2), *Rhodococcus* (1), and *Bacillus* (1) (Figure 1, Supplementary Table S2). It should be noted that *Pseudomonas* phage POR1 was further classified as a double-stranded DNA Siphovirus Dyson et al. (2016). The characterized viral CAZymes comprise four glycoside hydrolase (GH) family groups: GH43 (group 1), GH43/GH51/GH54/GH62/GH2/GH3 (group 2), GH43/GH30/GH39/GH51/GH52/GH54/GH1/GH116/GH120 (group 3), and GH23 (group 4) (Figure 3, Supplementary Table S3). Hits of subject IDs in the National Center for Biotechnology Information (NCBI) along with the complete description of CAZymes and their CAZY families in the recovered four groups are provided in Supplementary Table S4. The number and IDs of gene queries referring to all rhizosphere CAZymes across the four different kingdoms are given in Supplementary Table S5, while those for the four GH groups are given in Figure 3, Supplementary Tables S6–S7. The abundance of genes encoding all CAZymes in the two soil types is listed in Supplementary Table S8. The data provided in Supplementary Table S9 and described in Figure 4 indicate that the abundance of the seven characterized CAZymes was much higher in the rhizosphere microbiome than in the bulk soil microbiome.

Out of the 18 viruses of *M. oleifera* microbiomes, four viruses (*Mycobacterium* virus Babsiella, *Streptomyces* phage Lorelei, *Mycobacterium* virus Gaia, and *Streptomyces* phage Scap1) were proven to harbor genes encoding seven characterized CAZymes of CAZy GH class, while two viruses (*Rhodococcus* virus Pepy6 and *Mycobacterium* virus Gaia) had uncharacterized CAZymes of EC 3.2.1.- (Figure 4, Supplementary Table S3). Out of the seven characterized CAZymes, three CAZymes, namely, alpha-L-arabinofuranosidase (EC 3.2.1.55) of *Mycobacterium* virus Babsiella, beta-xylosidase (EC 3.2.1.37) of *Streptomyces* phage Lorelei, and chitinase (EC 3.2.1.14) of *Streptomyces* phage Scap1, exist in the KEGG pathway “Amino sugar and nucleotide sugar metabolism.” The first CAZyme belongs to GH group 2, while the second belongs to group 3 and the third belongs to GH group 4. The four other characterized CAZymes do not participate in KEGG pathways. Three of them, namely, endo-arabinanase (EC 3.2.1.99), galactan 1,3-beta-galactosidase (EC 3.2.1.145), and endo-beta-1,4-xylanase (EC 3.2.1.8), exist in *Mycobacterium* virus Gaia, while the fourth, CAZyme lysozyme type G (EC 3.2.1.17), exists in *Streptomyces* phage Scap1. The first three CAZymes belong to GH group 1, while the fourth belongs to GH group 4 (Supplementary Table S7).

**Figure 5:**
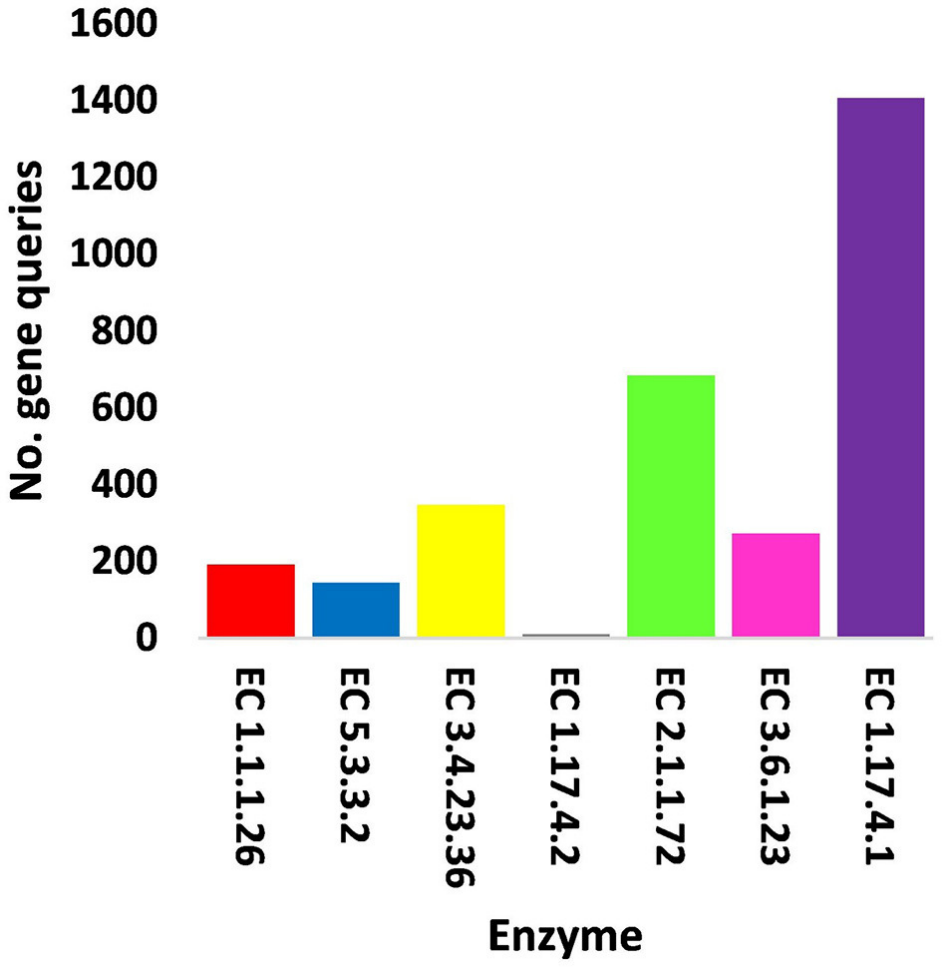
Query number of viral genes encoding enzymes generated from KEGG analysis in soil, e.g., bulk (S) and rhizosphere (R), microbiomes of *Moringa oleifera*. EC 1.1.1.26 = glyoxylate reductase, EC 5.3.3.2 = isopentenyl-diphosphate delta isomerase, EC 3.4.23.36 = signal peptidase II, EC 1.17.4.2 = ribonucleoside-triphosphate reductase (thioredoxin), EC 2.1.1.72 = DNA adenine methylase, EC 3.6.1.23 = dUTP pyrophosphatase, and EC 1.17.4.1 = ribonucleoside- diphosphate reductase alpha/beta chain. For more details, see Supplementary Table S13.

**Figure 6:**
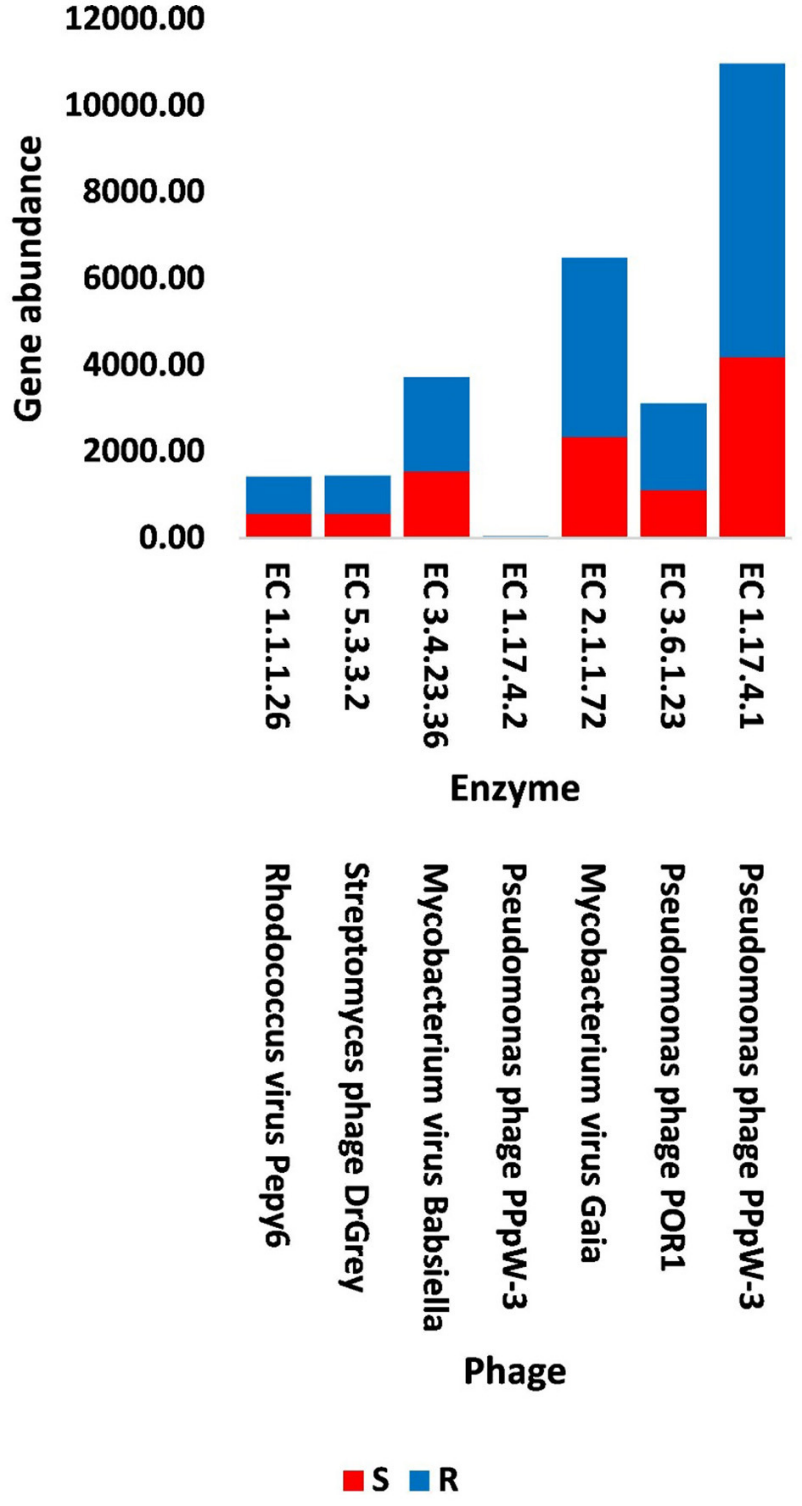
Abundance of viral genes encoding enzymes generated from KEGG analysis in soil, e.g., bulk (S) and rhizosphere (R), microbiomes of *Moringa oleifera*. EC 1.1.1.26 = glyoxylate reductase, EC 5.3.3.2 = isopentenyl-diphosphate delta isomerase, EC 3.4.23.36 = signal peptidase II, EC 1.17.4.2 = ribonucleoside-triphosphate reductase (thioredoxin), EC 2.1.1.72 = DNA adenine methylase, EC 3.6.1.23 = dUTP pyrophosphatase, and EC 1.17.4.1 = ribonucleoside- diphosphate reductase alpha/beta chain. For more details, see Supplementary Table S15.

In terms of other functioning enzymes in the virome of *M. oleifera* microbiomes, the results of the KEGG database indicated the participation of six viruses in the biosynthesis of seven enzymes (Supplementary Table S10). These viruses belong to tailed bacteriophages of class Caudoviricetes. The seven KEGG enzymes participate in six pathways, namely, “Glyoxylate and dicarboxylate metabolism,” “Terpenoid backbone biosynthesis,” “Protein export,” “Purine metabolism,” “Pyrimidine metabolism,” and “Mismatch repair.” KEGG IDs of these pathways are map00630, map00900, map03060, map00230, map00240, and map03430, respectively. Enzymes of the six pathways include glyoxylate reductase (EC 1.1.1.26) of *Rhodococcus* virus Pepy6 for pathway with ID map00630, isopentenyl-diphosphate delta isomerase (EC 5.3.3.2) of *Streptomyces* phage DrGrey for pathway with ID map00900, signal peptidase II (EC 3.4.23.36) of *Mycobacterium* virus Babsiella for pathway with ID map03060, ribonucleoside-triphosphate reductase (EC 1.17.4.2), ribonucleoside-diphosphate reductase subunit M1 (EC 1.17.4.1) of *Pseudomonas* phage PPpW-3 for pathways with IDs map00230 and map00240, DNA adenine methylase (EC 2.1.1.72) of *Mycobacterium* virus Gaia for pathway with ID map03430, and dUTP pyrophosphatase (EC 3.6.1.23) of *Pseudomonas* phage POR1 for pathway with ID map00240 (Supplementary Table S10). The full description of query IDs and their hits in the NCBI along with their participation in KEGG pathways are listed in Supplementary Table S11. The number of gene queries for all KEGG enzymes in the rhizosphere soil microbiome of *M. oleifera* is listed in Supplementary Table S12, while the number of viral gene queries for the seven KEGG enzymes across soil types is shown in Figure 5, Supplementary Table S13. The abundance of genes encoding all KEGG enzymes in microbiomes of the two soil types is given in Supplementary Table S14. The data provided in Supplementary Table S15 and described in Figure 6 indicate that the abundance of the seven CAZymes was higher in the rhizosphere microbiome than in the bulk soil microbiome. Overall, three phages harbor genes encoding members of CAZymes and other KEGG enzymes. They are *Mycobacterium* virus Babsiella, *Mycobacterium* virus Gaia, and *Rhodococcus* virus Pepy6 (Supplementary Tables S3, S10).

**Figure 7:**
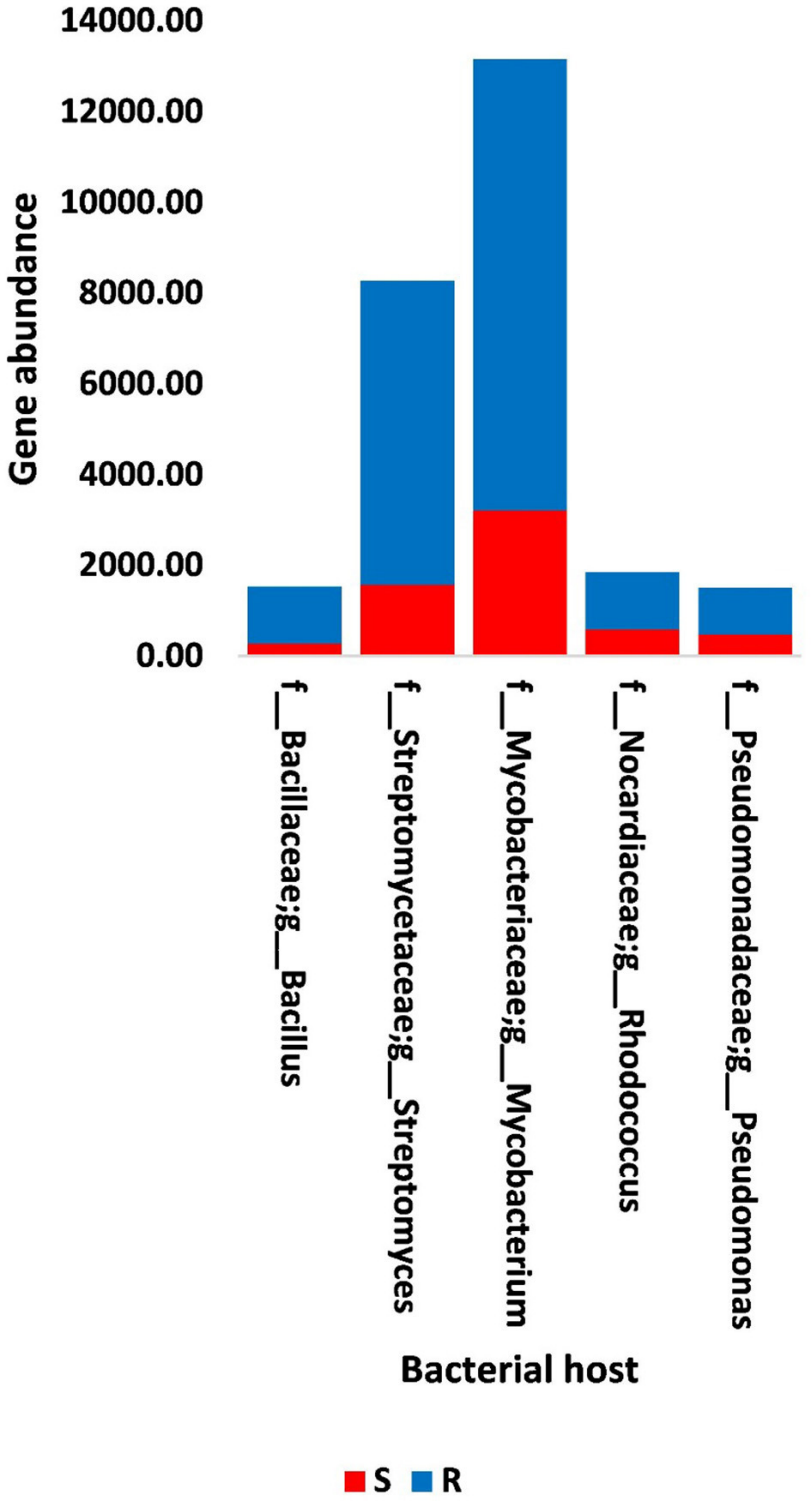
Abundance of genes in bacteria that host phages of soil, e.g., bulk (S) and rhizosphere (R), microbiomes of *Moringa oleifera*. For more details, see Supplementary Table S17.

**Figure 8:**
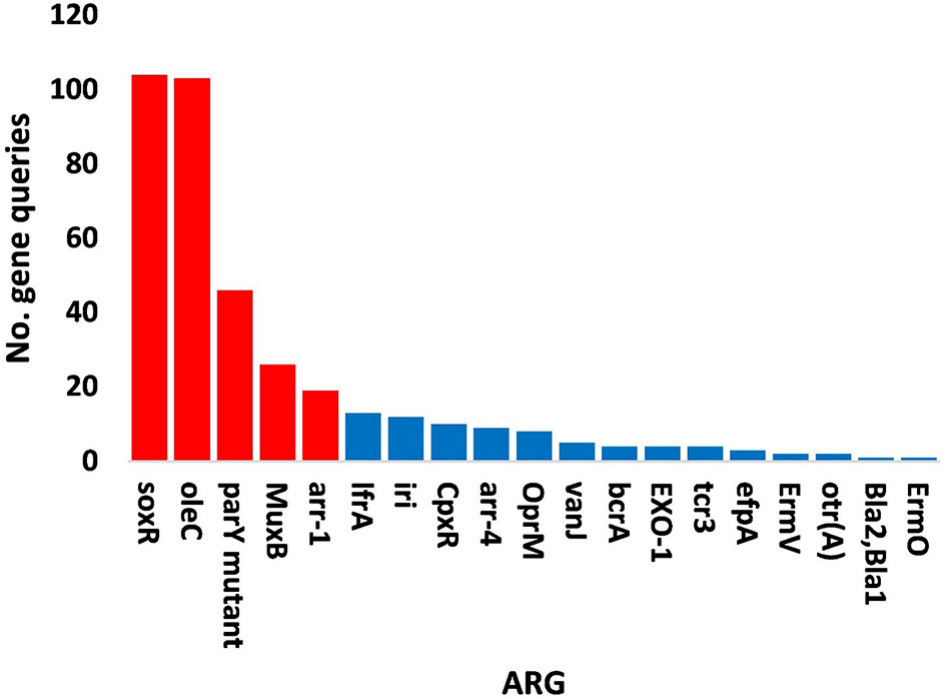
Query numbers of antibiotic resistance genes (ARGs) in the phageome of soil, e.g., bulk (S) and rhizosphere (R), microbiomes of *Moringa oleifera*. Columns in red refer to highly abundant ARGs to be utilized further. For more details, see Supplementary Table S20.

**Figure 9:**
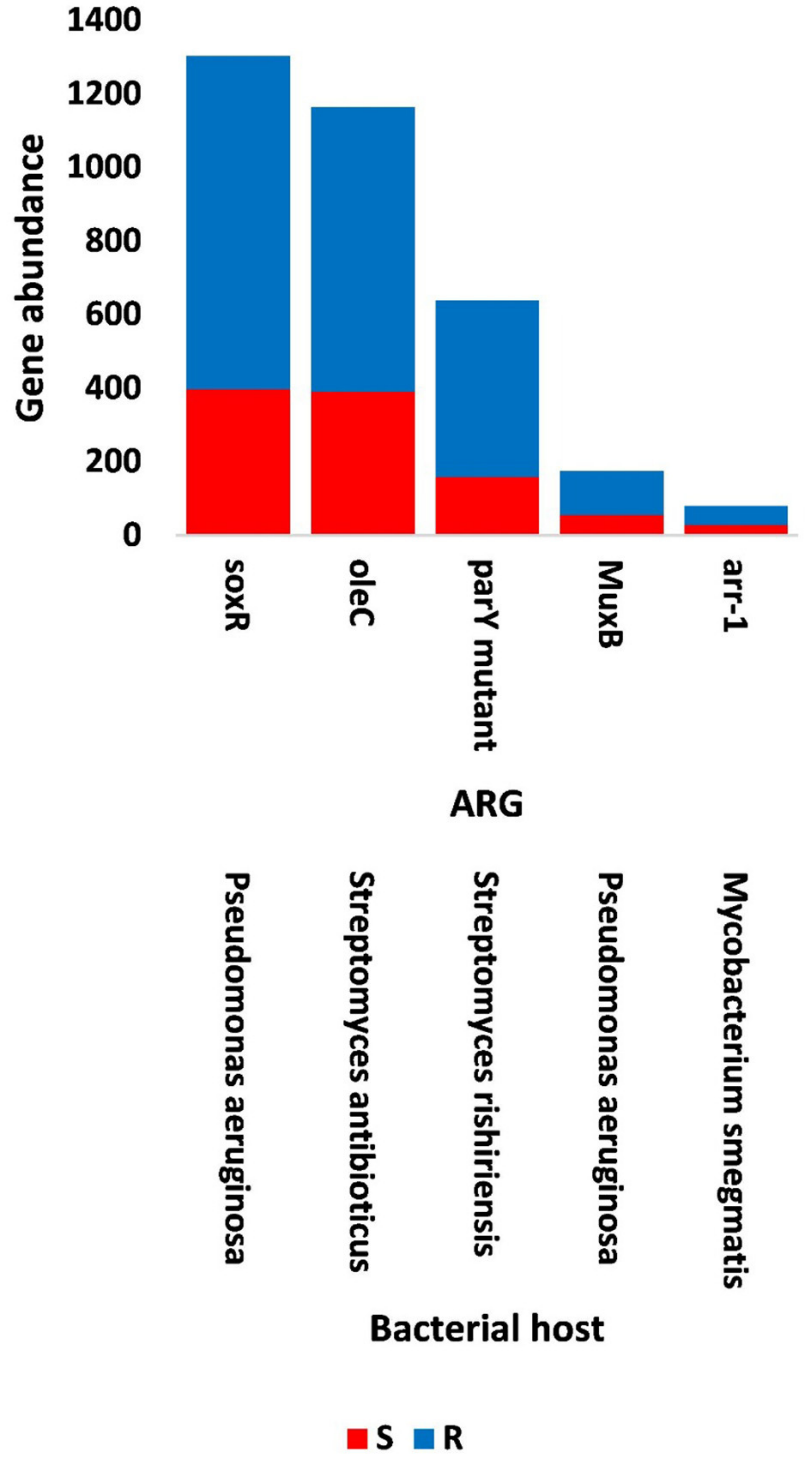
Abundance of the selected antibiotic resistance genes (ARGs) in the phageome of soil, e.g., bulk (S) and rhizosphere (R), microbiomes of *Moringa oleifera*. For more details, see Supplementary Table S22.

The results of bacterial abundance in the rhizospheric soil microbiome of *M. oleifera* and that of the surrounding bulk soil are given in Supplementary Table S16. The results for the five phage-associated bacterial genera, namely, *Pseudomonas, Streptomyces, Mycobacterium, Rhodococcus*, and *Bacillus*, indicated higher abundance of these bacterial genera in the rhizosphere soil than in bulk soil (Figure 7, Supplementary Table S17). ARGs that are encoded mostly by bacterial genes in soil microbiomes of *M. oleifera* are described in Supplementary Table S18. The number of gene queries of these ARGs is listed in Supplementary Table S19. The results showed that 19 ARGs were mostly encoded by phages within the five bacterial genera (Figure 8, Supplementary Table S20). The top five ARGs in terms of the number of gene queries were studied further. They exist in phages of three of the five bacterial genera, namely, *Pseudomonas, Streptomyces*, and *Mycobacterium*. These five ARGs are *soxR, oleC, parY* mutant, *MuxB*, and *arr-1*. The ARGs *soxR* and *MuxB* are encoded within *Pseudomonas aeruginosa* mostly by *Pseudomonas* phages PPpW-3 and/or POR1, the ARGs *oleC* and *parY* mutant are, respectively, encoded within *Streptomyces antibioticus* and *S. rishiriensis* mostly by *Streptomyces* phages Immanuel3, DrGrey, Lorelei, Mildred21 and/or Scap1, while the ARG *arr-1* is encoded within *Mycobacterium smegmatis* mostly by *Mycobacterium* virus Babsiella, Gaia, Dori, Edugator, Journey13, Kratio, Nerujay, Phasih and/or Squirty (Supplementary Table S2). These ARGs were highly abundant in the rhizosphere soil of *M. oleifera* compared with those in bulk soil (Figure 9, Supplementary Tables S21, S22). The description of the five ARGs along with their targeted antibiotics, resistance mechanisms, and antimicrobial resistance (AMR) gene families are given in Supplementary Table S23.

## 4. Discussion

Viruses are known to infect the three domains of life, namely, bacteria, archaea, and eukarya. The indirect influence of phages includes interaction with their bacterial hosts in order to promote a certain action, e.g., release certain compounds, which subsequently affects the ultimate Eukarya host (Pratama et al., 2020). Furthermore, rhizospheric bacteriophages have a great influence on soil microbial community (Emerson, 2019) as they regulate their structure, function, and diversity by lysing microbial cells and reprogramming metabolism processes via the expression of virus-encoded auxiliary metabolic genes (AMGs) (Braga et al., 2020; Luo et al., 2022). The latter actions can be beneficial or harmful to both soil microbes and intact plants based on the entity of the genes packaged in the virus.

The presence of non-viral genes in bacteriophages, likely occurring accidentally, is an intermediate step toward the horizontal gene transfer (HGT) and the spreading of one or more genes within a microbiome of a given host (Berglund, 2015). Horizontal transfer of bacterial genes via transformation, conjugation, and transduction to their analogs in the human pathogenic strains was speculated to be possible (Andersson and Hughes, 2010; Shami et al., 2022). The transduction of ARGs among bacteria via bacteriophages was almost neglected although phages are estimated to be the most abundant biological entities on Earth (Dion et al., 2020). The common transduction process is of a generalized type (Chen and Novick, 2009; Fillol-Salom et al., 2019), thus, occurred through accidental packaging (Wang et al., 2018). The latter action occurs by randomly incorporating bacterial genome fragments into phage capsids followed by subsequent transfer to genomes of other bacterial hosts. Thus, HGT among genetically related bacteria likely results in the exchange of genetic materials, such as ARGs, among different bacterial populations and their phages (Modi et al., 2013; Brown-Jaque et al., 2015).

### 4.1. CAZymes encoded by rhizospheric phageome

Horizontal gene transfer (HGT) can be beneficial in case of genes encoding CAZymes or other KEGG pathway enzymes, while raise concerns to human health in case of ARGs. Rhizosphere is known to receive large amounts of plant-assimilated carbon (C) mostly in the form of glucose through a functionally dynamic process called rhizodeposition (Hamonts et al., 2018; Pérez-Izquierdo et al., 2019). In turn, soil mineralization referring to C transformation is a process that involves microbial CAZymes (Pett-Ridge et al., 2021). In the present study, a number of viral genes were shown to promote the biosynthesis of CAZymes and metabolites in bacteria (Figures 4, 6, Supplementary Tables S3, S10) with an indirect influence on the plant host. Aligning with the results of the present study, CAZymes of the GH23 family were previously proven to be encoded by bacteria and by a bacteriophage (https://www.cazypedia.org/index.php/Glycoside_Hydrolase_Family_23) (Blackburn and Clarke, 2001).

Carbohydrate-Active enZymes belong to CAZy classes that are subclassified into families with a range of discrete modules acting on building and/or degrading complex carbohydrates (Cantarel et al., 2009). CAZy classes include class glycoside hydrolases (GHs) that mostly act on the hydrolysis and/or rearrangement of glycosidic bonds (Lombard et al., 2014). The most abundant GH CAZy families in the rhizosphere virome of *M. oleifera* are GH43 with nine CAZymes and GH23 with two CAZymes (Supplementary Tables S3, S4, S7). Based on the family composition of these 11 CAZymes, they were separated into four GH CAZyme groups (1, 2, 3, and 4). Three of these GH groups involve CAZymes from the GH43 family and a few other GH families, while the fourth group involves the GH23 family (Figure 3, Supplementary Table S7). CAZymes of the GH43 CAZy family are among the largest GH families (Mewis et al., 2016) that act on debranching and degradation of hemicellulose (e.g., arabinoxylans) and pectin polymers albeit displaying a hitherto underestimated variety of discrete specificity features as its CAZymes might require more than one substrate for their optimal activity (Romeis et al., 1993). Members of the GH23 family are lytic transglycosylases or peptidoglycan lyases that require the peptide side chains in peptidoglycan for their activity, while are inactive against chitin (Romeis et al., 1993). In terms of its member lysozyme type G, earlier reports indicated a typical inverting mechanism of action (Helland et al., 2009).

Four of the GH43 CAZymes (with EC 3.2.1.-) were not further studied as they were not fully characterized. Three of the seven, fully characterized CAZymes, namely alpha-L-arabinofuranosidase (EC 3.2.1.55) of *Mycobacterium* virus Babsiella, beta-xylosidase (EC 3.2.1.37) of *Streptomyces* phage Lorelei, and chitinase (EC 3.2.1.14) of *Streptomyces* phage Scap1, were shown to exist in the KEGG pathway “Amino sugar and nucleotide sugar metabolism” with ID map00520 (Supplementary Figure S1). The first CAZyme belongs to GH group 2 and participates in the conversion of arabinan to L-arabinose that is eventually converted into two UDP sugars (UDP-L-arabinose and UDP-D-xylose), while the second CAZyme belongs to group 3 and participates in the conversion of the latter metabolite into X-xylose. The combined action of these two CAZymes mediates the passage to the pathway “Pentose and glucuronate interconversion” (Supplementary Figure S1). Another CAZyme of *Mycobacterium* virus Gaia namely endo-arabinanase (EC 3.2.1.99) that belongs to GH group 1 (Supplementary Table S7) also participates in the degradation of arabinan, which mediates the action of alpha-L-arabinofuranosidase (EC 3.2.1.55) (Shi et al., 2014). The resulted L-arabinose residues accumulate in pectins of the plant cell wall (Sakamoto et al., 2003). Thus, the two *Mycobacterium* phages act synergistically via the action of the two CAZymes with ECs 3.2.1.55 and 3.2.1.99. The third CAZyme of the KEGG pathway “Amino sugar and nucleotide sugar metabolism” namely chitinase (EC 3.2.1.14) participates in the conversion of chitin into N-acetylglucosamine (GlcNAc). This metabolite is an amide derivative of glucose and a part of a biopolymer of the bacterial cell wall, which cross-links with MurNAc to recover the important layered cell wall structure of peptidoglycan. GlcNAc is also a main component of the fungi cell wall (Kamel et al., 1991). The GH group 1 CAZymes, such as galactan 1,3-beta-galactosidase (EC 3.2.1.145) and endo-beta-1,4-xylanase (EC 3.2.1.8), are encoded by the genes of *Mycobacterium* virus Gaia. The first CAZyme belongs to type II arabinogalactans (AGs)-degrading enzymes (Sakamoto et al., 2011). AG proteins promote several physiological events in the plant such as cell–cell recognition, embryogenesis, and programmed cell death (PCD) (Gaspar et al., 2001). The second CAZyme is responsible for the hydrolysis of β-1,4 bonds in plant xylan, which is the major component of hemicellulose of plant cell walls (Zeng et al., 2017). Thus, plant cells seem to differentially promote the abundance level of the hosting bacterium *Mycobacterium* and its associated phages based on their requirements. The GH group 4 lysozyme type G (EC 3.2.1.17) of *Streptomyces* phage Scap1 is among the three types of lysozymes that act as antibacterial compounds where they breakdown the peptidoglycan of bacterial cell walls and therefore induce bacterial cell lysis (Oliver and Wells, 2015; Nawaz et al., 2022). This viral action seems to be selective and based on microbe and plant cell requirements.

### 4.2. Other rhizospheric KEGG enzymes encoded by phageome

In terms of other KEGG enzymes encoded by viral genes, glyoxylate reductase (1.1.1.26) of *Rhodococcus* virus Pepy6 was shown to exist in the KEGG pathway “Glyoxylate and dicarboxylate metabolism” with ID map00630 (Supplementary Figure S2). This enzyme participates in the conversion of the reactive oxygen species (ROS) namely hydrogen peroxide (H_2_O_2_) to oxygen (O_2_) as an intermediate step toward the conversion of glycolate to glyoxylate, which is the core step of the pathway (Supplementary Figure S2). Thus, the virus might help reduce the level of H_2_O_2_ when reached a certain threshold. The latter reaction can be followed by the biosynthesis of the two important metabolites glycine and malate (Supplementary Figure S2). Reactions of the glyoxylate cycle were proven to promote the permeability of the peroxisome membrane (Kunze and Hartig, 2013). The enzyme also participates in the conversion of hydroxyl-pyruvate to glycerate that can eventually promote carbon fixation in plants (Supplementary Figure S2). The enzyme isopentenyl-diphosphate delta isomerase (EC 5.3.3.2) of *Streptomyces* phage DrGrey participates in the KEGG pathway “Terpenoid backbone biosynthesis” (Supplementary Figure S3). It catalyzes the conversion of the less-reactive isopentenyl pyrophosphate (IPP) to the more-reactive electrophile dimethylallyl pyrophosphate (DMAPP). This reaction is considered a rate-limiting step in the biosynthesis of isoprenoids through the mevalonate and the MEP pathways (Supplementary Figure S3). The downstream step involves the biosynthesis of isoprene, whose emission is positively correlated with temperature toward protecting plants from heat stress (Rodrigues et al., 2020). The enzyme signal peptidase II (EC 3.4.23.36) of *Mycobacterium* virus Babsiella participates in the KEGG pathway “Protein export” (Supplementary Figure S4). It catalyzes the release of signal peptides from bacterial membrane prolipoproteins. This Sec-dependent pathway refers to the active transport of bacterial proteins across the cell membrane or to the periplasmic cell compartment (Rusch and Kendall, 2007). These actions are mediated by signal peptides that are responsible for targeting the respective proteins by the membrane-bound Sec translocase (Rusch and Kendall, 2007).

The DNA adenine methylase or Dam (EC 2.1.1.72) of *Pseudomonas* phage PPpW-3 is important when bacterial cells require making mismatch repair. When DNA polymerase III generates a mismatched base-pair(s) during DNA synthesis, then the cell can differentiate between the template methylated strand and the newly synthesized (daughter) unmethylated strand by the action of Dam (Barras and Marinus, 1989). Then, repair complex MutS/MutL activates the endonuclease MutH that binds the hemimethylated site and selectively cleaves the unmethylated daughter strand to promote the excision of the mismatch portion of the nascent strand by exonuclease and re-synthesis by DNA polymerase III (Supplementary Figure S5) (Barras and Marinus, 1989; Løbner-Olesen et al., 2005). In addition, methylation status by Dam epigenetically manipulates bacterial RNA transcription (Casadesús and Low, 2006) and was also proven to promote bacterial cell viability (Julio et al., 2001).

The ribonucleoside-triphosphate reductase (EC 1.17.4.2) and ribonucleoside-diphosphate reductase alpha/beta chain (EC 1.17.4.1) of *Pseudomonas* phage PPpW-3 participate in the two pathways “Purine metabolism” (Supplementary Figure S6) and “Pyrimidine metabolism” (Supplementary Figure S7). These enzymes are essential in the conversion of ribonucleotides to deoxyribonucleotides, e.g., building blocks for DNA replication (Torrents, 2014). Therefore, these two enzymes affect the downstream steps of cell division by the balanced supply of dNTPs, which results in higher mutation rate and genome instability when the dNTPs rate of production is imbalanced (Mathews, 2006). To support successful DNA replication, the enzyme dUTP pyrophosphatase (EC 3.6.1.23) of *Pseudomonas* phage POR1 catalyzes the hydrolysis of dUTP to both dUMP and pyrophosphate to prevent the incorporation of uracil into DNA during replication (Supplementary Figure S7), thus, save energy required for proof-reading and repair. In addition, the incorporation of uracil in DNA strands promotes bacterial cell death; a case of thymine-less death (Mathews, 2006).

### 4.3. ARGs of rhizospheric phageome

The top five highly abundant ARGs in the rhizosphere of *M. oleifera* were shown to be encoded by the phages of bacterial genera *Pseudomonas, Streptomyces*, and *Mycobacterium* (Figure 9, Supplementary Table S22). Of which, *Pseudomonas* is within the bacterial group namely ESKAPE (*Enterococcus, Staphylococcus, Klebsiella, Acinetobacter, Pseudomonas*, and *Escherichia*) that poses concerns of resistance to antibiotics in genetically related pathogens (Boucher et al., 2009). Candidate ARGs of *Pseudomonas* in the present study include *soxR* and *MuxB* (Figure 9).

Rhizospheric resistomes of naturally growing wild plant species might contain ARGs with unexplored antibiotic resistance mechanisms (Berendonk et al., 2015; Obermeier et al., 2021). ARGs of such soil resistomes might pose a direct threat to human health due to HGT to human genetically related pathogenic microbes if bacteria harboring these ARGs are in direct contact with an edible plant such as *M. oleifera*. Based on the results of the present study, abundant mechanisms of antibiotic resistance in the rhizosphere of *M. oleifera* include “antibiotic efflux pump” for ARGs *soxR, OleC*, and *MuxB*, “antibiotic target alteration” for *parY mutant*, and “antibiotic inactivation” for *arr-1* (Supplementary Table S23). Antibiotic efflux mechanisms of the *soxR* gene include AMR gene families “major facilitator superfamily (MFS),” “ATP-binding cassette (ABC),” and “resistance-nodulation-cell division (RND),” while the efflux mechanism of the *oleC* gene includes ATP-binding cassette (ABC) and that of *MuxB* gene includes resistance-nodulation-cell division (RND) (Supplementary Table S23).

#### 4.3.1. Efflux pump resistance mechanism in the phageome of *M. oleifera*

The efflux pump is an energy-dependent mechanism that promotes bacterial homeostasis via the expulsion of toxic substances or antibiotics (Fernández and Hancock, 2012). This activity reduces the abundance of antibiotics in the cell. This mechanism is favored by bacterial pathogens to promote survival during the infectivity period. Thus, this mechanism of expelling toxins should be faster than host-responsive defense mechanisms (Koprivnjak and Peschel, 2011). Efflux pump families are made of five AMR families. The most common is the resistance-nodulation-division (RND), followed by the ATP-binding cassette (ABC) and the major facilitator superfamily (MFS) (Piddock, 2006; Poole, 2007; Sun et al., 2014). The RND family of efflux pump mechanism uses proton motive forces for efflux (Chitsaz and Brown, 2017) and is only found in gram-negative bacteria, such as *Pseudomonas* with *soxR* and *MuxB* genes (Figures 8, 9), whereas the ATP-binding cassette (ABC) family relies on ATP hydrolysis and can also be found in gram-positive bacteria (Sun et al., 2014), such as *Streptomyces* with *oleC* gene (Figures 8, 9).

The *soxR* gene is a transcription factor that regulates bacterial growth, fitness, and resistance against several antibiotics such as tetracycline, penam, cephalosporin, and rifamycin antibiotics (Palma et al., 2005; Li et al., 2017). This gene also promotes several other efflux pump genes of the RND family (Marchand et al., 2004; Gu and Imlay, 2011). Two oxidation reactions in a raw take place upon the exposure of bacteria and its phages to a given antibiotic. The first reaction involves SoxR protein, while the second involves another protein namely SoxS. This oxidation system is called the SoxR/SoxS paradigm (Pomposiello and Demple, 2001; Li et al., 2017), of which their association promotes the overexpression of a multidrug efflux machinery namely AcrAB-TolC; a tripartite transporter that expels intracellular periplasm substrates (White et al., 1997; Ruiz and Levy, 2014). In the absence of SoxS protein, SoxR ought to drive a 6-gene regulon that promotes efflux pump in members of the Proteobacteria, such as *Pseudomonas aeruginosa* (Bialek-Davenet et al., 2011; Li et al., 2017). The latter approach seems to be the ideal efflux mechanism promoted by the phageome of *P. aeruginosa* in the present study. SoxR also facilitates the induction of the major facilitator superfamily (MFS) efflux pump genes (Saidijam et al., 2006; Dulyayangkul et al., 2016). The MFS system contains transport proteins or facilitators that force unwanted solutes to move across membranes (Marger and Saier, 1993). Thus, we expect that the viral *soxR* gene can pose a major risk to human health, through the driving expression of the 6-gene regulon in *Pseudomonas aeruginosa*, if this gene is horizontally transferred to the human gut microbiome. In terms of the ARG *muxB*, it was reported that it encodes a component of the MuxABC-OpmB efflux pump against several antibiotics including tetracycline and aminocoumarin (Mima et al., 2009). This pump is the last hitherto detected RND-type multidrug efflux pump in *P. aeruginosa*. Unlike other RND-type efflux pumps, the MuxABC-OpmB pump includes two RND components (MuxB and MuxC), in addition to one MFP component (MuxA) and one OMP component (OpmB). Interestingly, the ARG *cpxR* was reported to enhance the expression of genes encoding *mexAB-oprM* efflux pump, thus, also enhancing resistance against aminocoumarin and tetracycline antibiotics. The gene encoding CpxR was proven to be moderately abundant in the rhizospheric phageome of *M. oleifera* (Figure 8, Supplementary Table S21).

The ATP-binding cassette (ABC) antibiotic efflux pump of *Streptomyces* viral gene *oleC* is made of membrane and membrane-associated (e.g., AAA ATPases) proteins that expel antibiotics out of the bacterial cell membranes. OleC transporter protein of *Streptomyces antibioticus* is made of two proteins (Olano et al., 1996). The first, namely OleC, can bind/hydrolyze ATP, while the second, namely OleC5, is a hydrophobic membrane protein. Through the ABC transporter system of the viral *oleC* gene, the antibiotic oleandomycin is effluxed as a self-resistance mechanism against this antibiotic (Ma Rodriguez et al., 1993). More importantly, bacterial genus *Streptomyces* was reported to harbor ARGs that encode the majority of the clinical antibiotics (e.g., neomycin, cypemycin, grisemycin, and chloramphenicol), and to act as a factory of newly emerged antibiotics. However, invasive infection with this bacteria in clinical practice is fortunately rare (Kapadia et al., 2007). The present study speculates that the portion of the ARGs of genus *Streptomyces* might exist in its phages for storage or as an intermediate step toward the dynamic transfer to other genetically related bacteria.

#### 4.3.2. Antibiotic target alteration and inactivation resistance mechanisms in the phageome of *M. oleifera*

In terms of the antibiotic target alteration mechanism of *parY mutant*, prior studies indicated that bacterial topoisomerase IV (topo IV) or type II topoisomerase is made of ParX and ParY subunits. The first subunit contains the catalytic center for DNA cleavage/rejoining, while the second contains the catalytic center for ATP hydrolysis. In *Streptomyces coelicolor*, the topoisomerase enzyme confers aminocoumarin antibiotic resistance. The mode of action of this antibiotic is inhibiting the drug target ParY of type II topoisomerase, such as gyrase (Maxwell, 1997). *Streptomyces* species have the ability to resist this antibiotic by *de novo* synthesizing a modified B subunit of gyrase, namely aminocoumarin-resistant gyrase B. Thus, when phages harboring this version of the *parY* gene transduce bacteria, it can inhibit the detrimental effects of aminocoumarin by altering the structure of gyrase B (Schmutz et al., 2004). In addition, there is another version of the resistance gene, namely *parY*^R^ that participates in encoding an enzyme that is basically not a target for aminocoumarin antibiotics.

**Figure 10:**
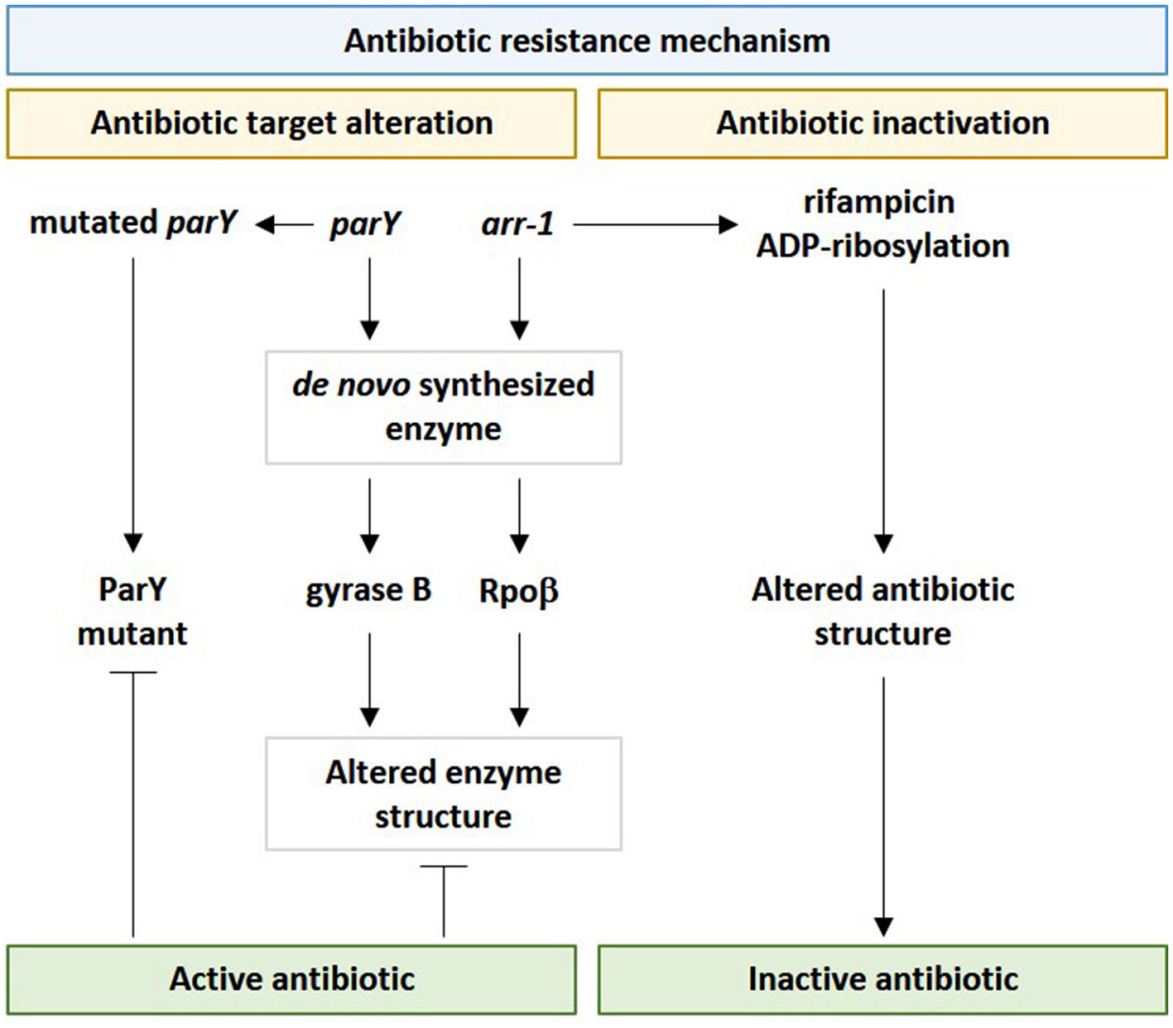
Influence of viral *parY* and *arr-1* genes in contributing antibiotic resistance in genus *Streptomyces* in the rhizospheric microbiome of *M. oleifera* via the two resistance mechanisms namely “antibiotic target alteration” and “antibiotic inactivation.” *parY* mutated gene acts against aminocoumarin, while *arr-1* gene acts against rifampicin.

Among bacterial resistomes, a new nomenclature, such as “enzystome” (Swaminath et al., 2020), was given to the large battery of bacterial enzymes and their mutants that implement antibiotic resistance mechanisms (Egorov et al., 2018). Rifampin ADP-ribosyltransferase (Arr) is among enzystome serving in antibiotic inactivation due to the action of the encoding gene *arr-1* of *Mycobacterium smegmatis*. Rifampin ADP-ribosyltransferase acts in catalyzing the ADP-ribosylation of rifampicin and other rifamycins, thus, inactivating them (Figure 10) (Morgado et al., 2021). Rifampicin mainly acts in binding the B subunit of the RNA polymerase (e.g., RpoB) to inhibit the transcription initiation of bacterial genes and to facilitate the direct blocking of the elongating RNA (Albano et al., 2019). A mutant version of the enzyme Arr was first emerged as a contributor to the resistance mechanism of “antibiotic target alteration,” as explained earlier for the *parY* gene. However, “antibiotic inactivation” or enzymatic antibiotic modification, such as ADP-ribosylation, is a resistance mechanism that modifies the structure of the antibiotic, not its target in the bacteria. Antibiotic inactivation further emerged as another resistance mechanism contributed by this enzyme. In terms of the ARG namely RpoB, inactivation of rifampicin by modifying its binding affinity so that it cannot bind bacterial RNA polymerase (Figure 10). This bacterial response is mediated by the enzymatic inactivation of the antibiotic by hydrolysis or by the formation of inactive forms of the antibiotic (Davies, 1994) as well as by the transfer of a chemical group, such as acetyl, phosphoryl, and adenyl, to the antibiotic by transferases (Reygaert, 2018). Acetylation is an effective approach against aminoglycosides, chloramphenicol, streptogramins, and fluoroquinolones, while phosphorylation and adenylation are effective against aminoglycosides (Blair et al., 2015). It was anticipated that the faster the inactivation process of the antibiotic upon entering the bacterial cell, the higher the chance for the bacteria to survive. As the *arr-1* gene originally exists in the bacterial chromosome, thus, this gene cannot be incorporated into phages except by accidental packaging (Wang et al., 2018); a phenomenon that might lessen the diversity of soil resistome and unify its bacterial genetic constituent.

In general, we support previous reports indicating that the soil bacteriome uses phageome in disseminating ARGs (Balcázar, 2018), and that phageome can promote the emergence of antibiotic-resistant pathogenic bacteria in the human gut via unintended horizontal transfer via this or other mobile genetic elements (MGEs) (Chen et al., 2017, 2019; Khan et al., 2018). Newly emerged ARGs can be the result of gene mutation or exchanges of MGEs between soil and clinical gut resistomes (Cheng et al., 2012; Forsberg et al., 2014). As indicated earlier, some reports support the occurrence of ARGs in phages due to accidental packaging prior to transduction (Wang et al., 2018). Examples include ARGs encoding the antibiotics lactamase (Brown-Jaque et al., 2015), quinolone (Colomer-Lluch et al., 2014), and vancomycin (Lekunberri et al., 2017) that were discretely detected in viral DNA (Moon et al., 2020). Furthermore, virome analyses, via whole-metagenomic shotgun sequencing, in several environments, such as oceans, freshwater, and clinical samples, indicated the occurrence of a large number of novel ARGs (Subirats et al., 2016). Phage-associated ARGs exist in clinical phageome due to the heavy exposure of bacteria and their phages to antibiotics (Colomer-Lluch et al., 2011; Brown-Jaque et al., 2015), thus, it is likely that ARGs within bacteria are also carried by phages in nature. Modi et al. (2013) proved that phages can further mediate ARG transduction between genetically related bacterial cells.

In conclusion, the present study provides information on the rhizospheric phageome signature of the wild plant *M. oleifera* indicating metabolic benefits to both bacteria and its interacting plant on the one hand, and the hazardous effects of disseminating soil antibiotic resistance genes (ARGs) in human pathogens or clinical isolates on the other hand. We recommend studying soil phageomes of other wild plants and those of soil compartments other than the rhizosphere in order to reach a better conclusion on the influence of phageomes in editing soil microbiomes and the consequent cross-talking pattern with the intact plant.

## Data availability statement

The data presented in the study are deposited in the European Nucleotide Archive (ENA) (https://www.ebi.ac.uk/ena/browser/) repository under project accession number PRJEB55112 and study accession number ERP139990. Sample accession numbers are ERR10100770, ERR10100772, ERR10100773, ERR10100774, ERP10100771, and ERP10100781.

## Author contributions

RA, RJ, and AA: conceptualization. RA, HS, MA, FS, and AA: methodology. SA, FA, HA, and RA: software. RA, LB, MT, IH, and MR: validation. RA, MR, IH, MT, LB, and AA: formal analysis. RA, HA, FA, and RJ: investigation. SA, RA, FS, and MA: resources. HS, RA, and RJ: data curation. RA and AA: writing—original draft preparation. RA, MA, MR, IH, MT, and LB: writing review and editing. RA, AA, HA, FS, HS, FA, and SA: visualization. AA: project administration and supervision. HS: funding acquisition. All authors read and agreed to the published version of the manuscript.
