# Supplementary material for: Functional annotation of rhizospheric phageome of the wild plant species Moringa oleifera
Source: Front Microbiol. 2023 May 16;14:1166148. doi: 10.3389/fmicb.2023.1166148 (PMC10227523; doi:10.3389/fmicb.2023.1166148)
Supplement: Supplementary file 1 [file Data_Sheet_1.zip › Supplementary Figures.docx]

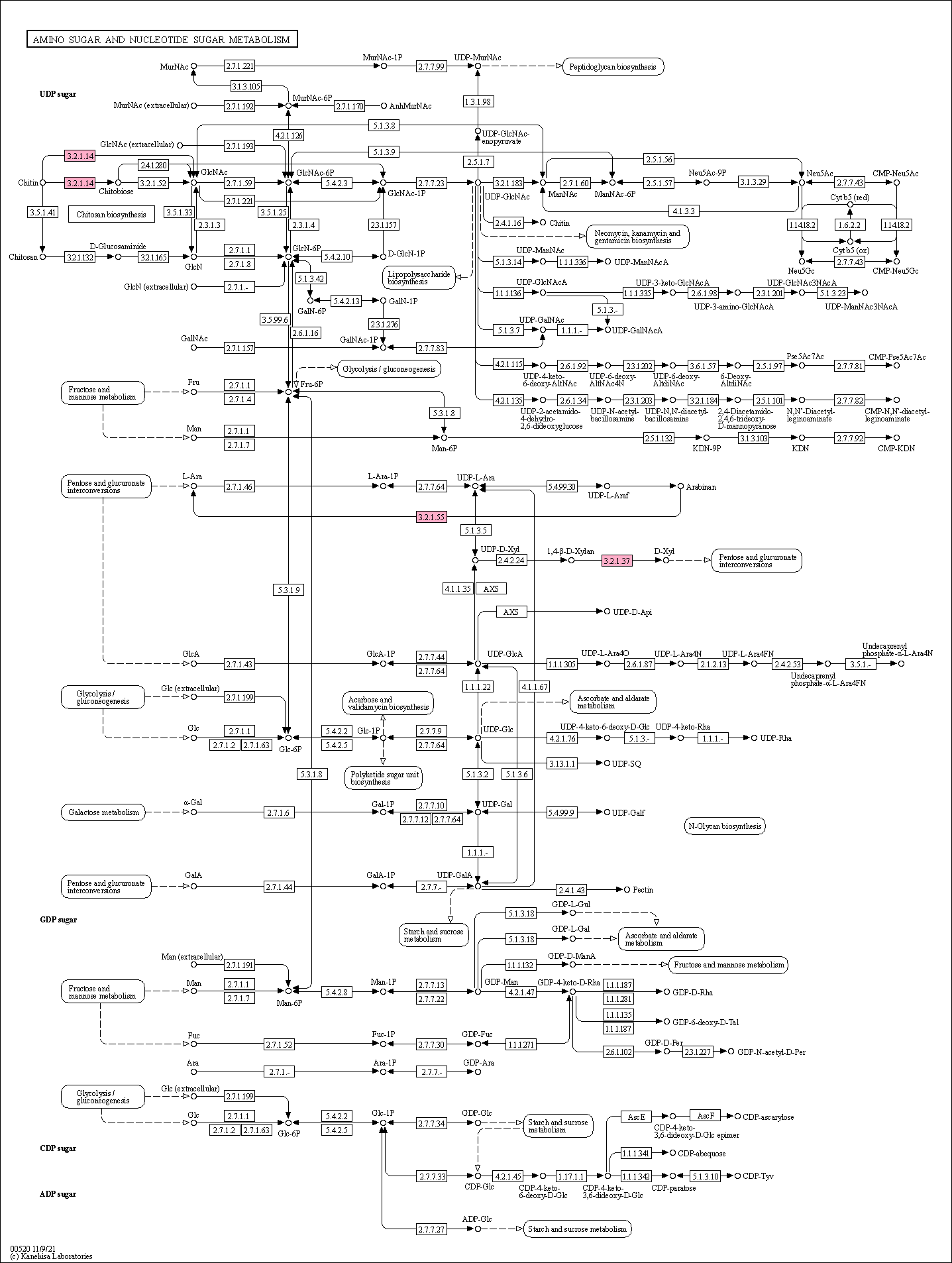
Figure S1. KEGG pathway “Amino sugar and nucleotide sugar metabolism” of sub-category “Carbohydrate metabolism” and category “Metabolism” referring to the three enriched CAZymes in rhizosphere virome of *M. oleifera*, e.g., alpha-L-arabinofuranosidase (EC 3.2.1.55), beta-xylosidase (EC 3.2.1.37), and chitinase (EC 3.2.1.14). Abundance of these three CAZymes in the two soil type is shown in Table S9.


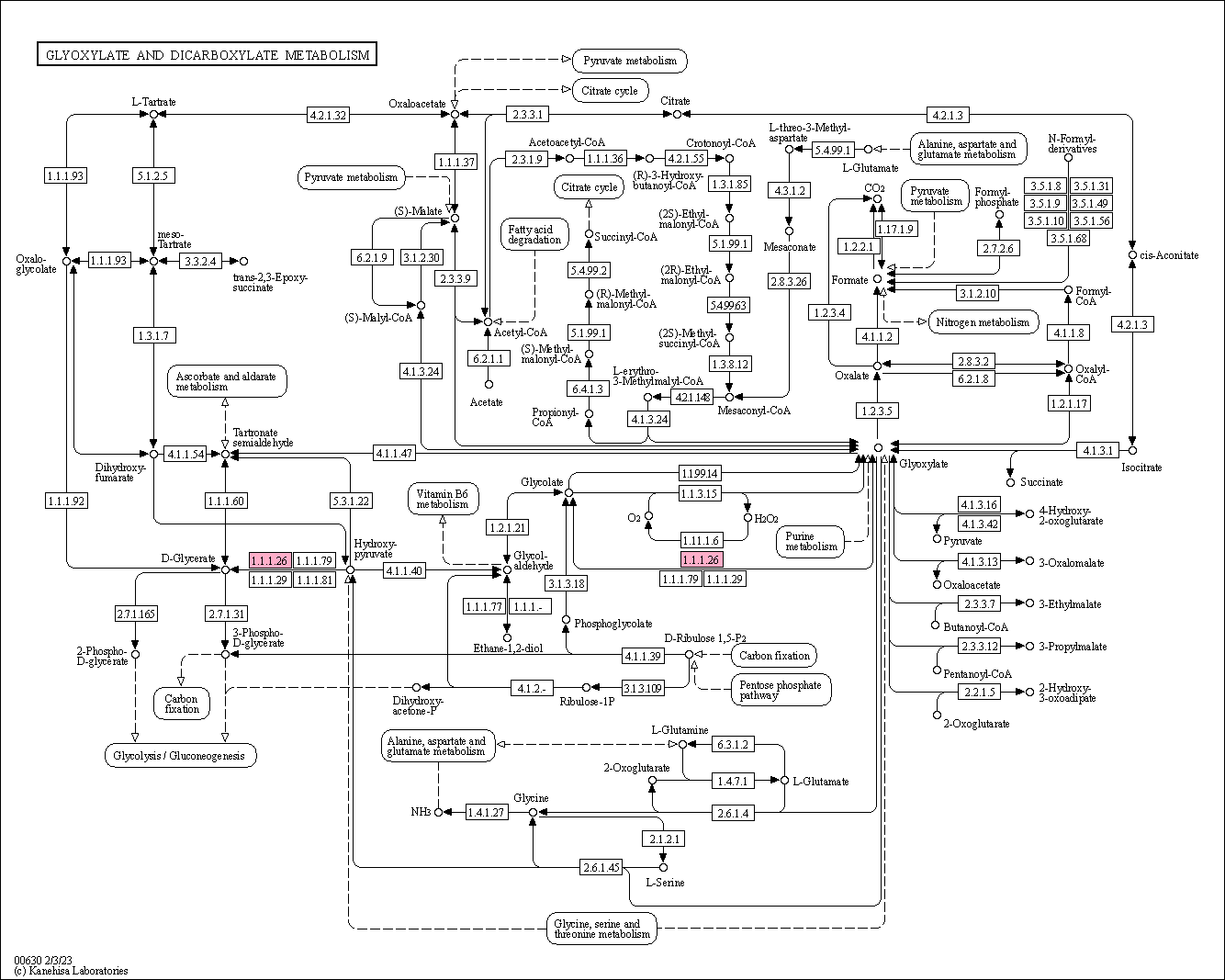
Figure S2. KEGG pathway “Glyoxylate and dicarboxylate metabolism” of sub-category “Carbohydrate metabolism” and category “Metabolism” referring to the enriched enzyme in rhizosphere virome of *M. oleifera*, e.g., glyoxylate reductase (EC 1.1.1.26). Abundance of this enzyme in the two soil type is shown in Table S15.

Figure S3. KEGG pathway “Terpenoid backbone biosynthesis” of sub-category “Metabolism of terpenoids and polyketides” and category “Metabolism” referring to the enriched enzyme in rhizosphere virome of *M. oleifera*, e.g., isopentenyl-diphosphate Delta-isomerase (EC 5.3.3.2). Abundance of this enzyme in the two soil type is shown in Table S15.
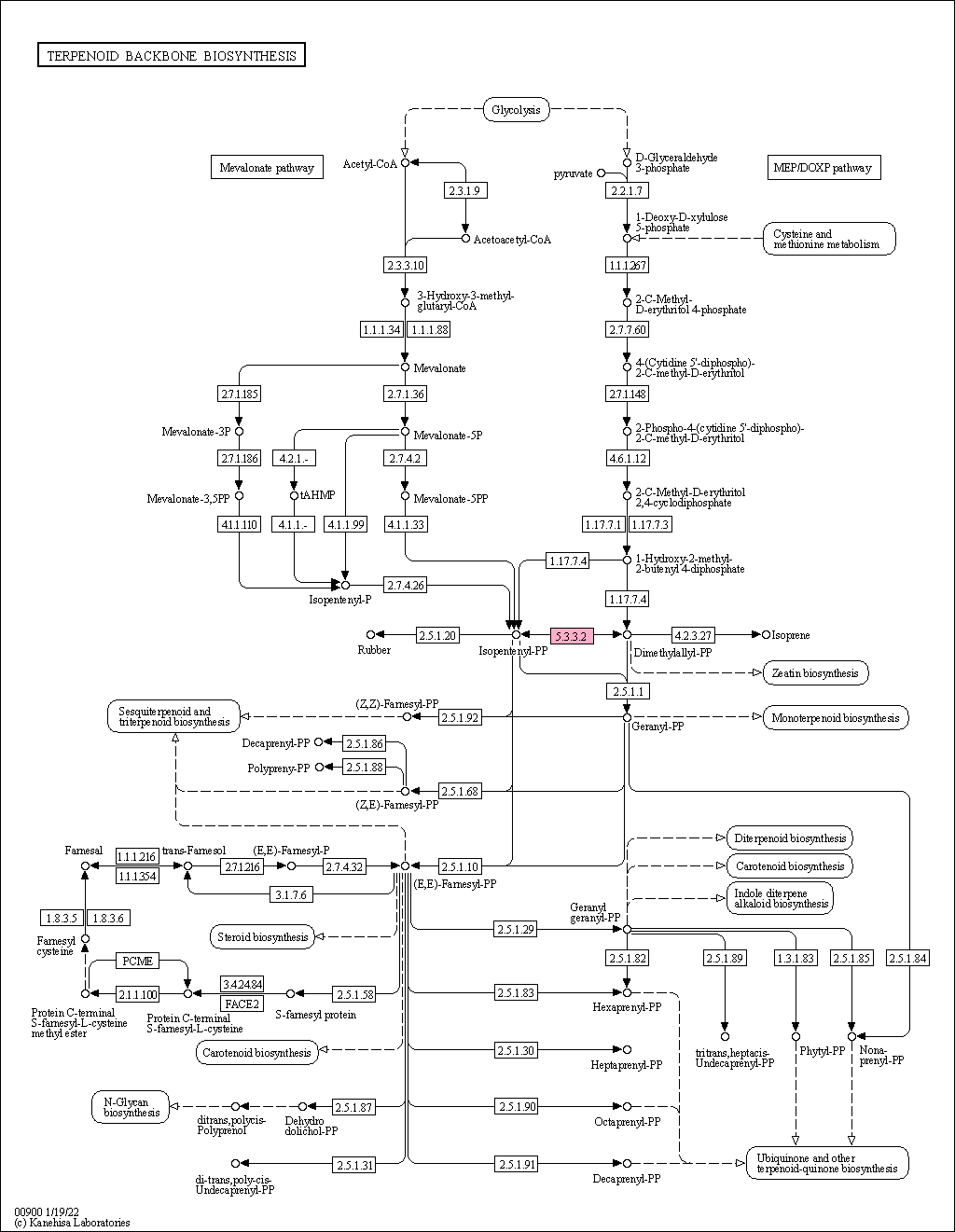


Figure S4. KEGG pathway “Protein export” of sub-category “Folding, sorting and degradation” and category “Genetic Information Processing” referring to the enriched enzyme in rhizosphere virome of *M. oleifera*, e.g., signal peptidase II (EC 3.4.23.36). Abundance of this enzyme in the two soil type is shown in Table S15.
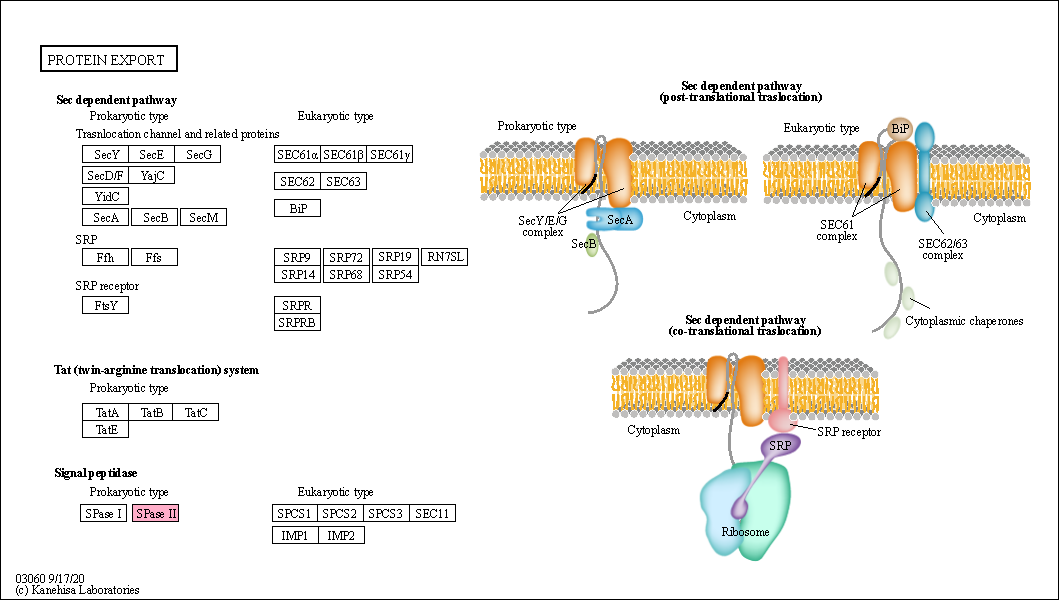


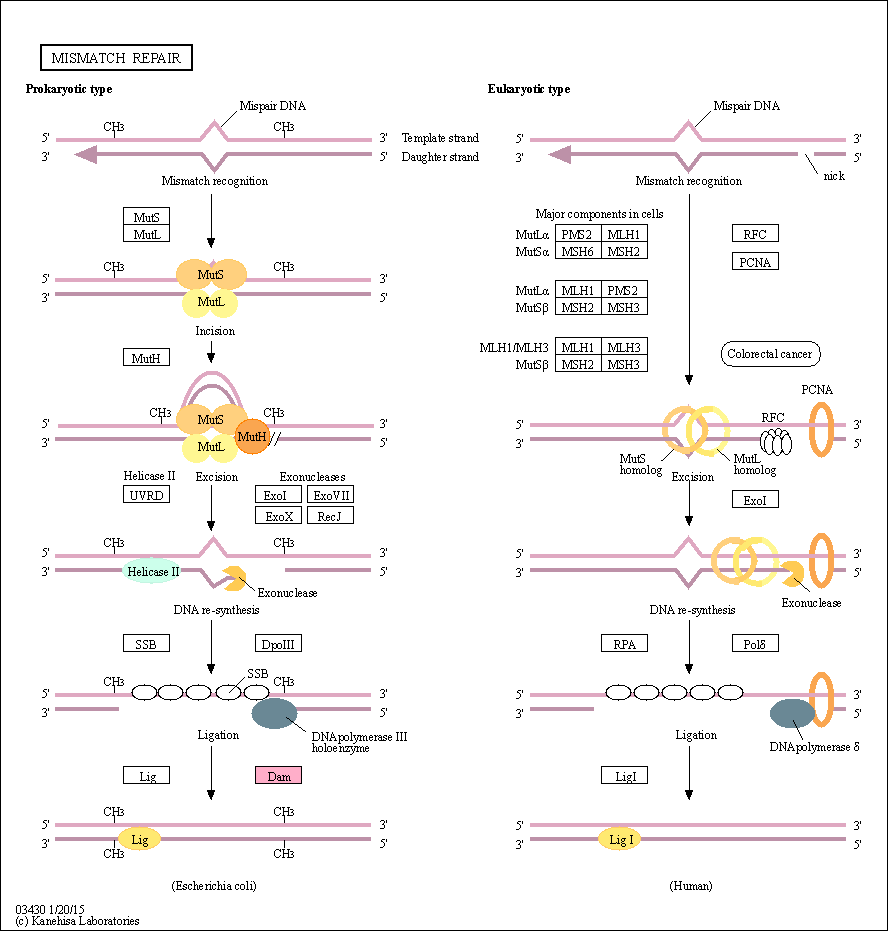
Figure S5. KEGG pathway “Mismatch repair” of sub-category “Replication and repair” and category “Genetic Information Processing” referring to the enriched enzyme in rhizosphere virome of *M. oleifera*, e.g., DNA adenine methylase (EC 2.1.1.72). Abundance of this enzyme in the two soil type is shown in Table S15.


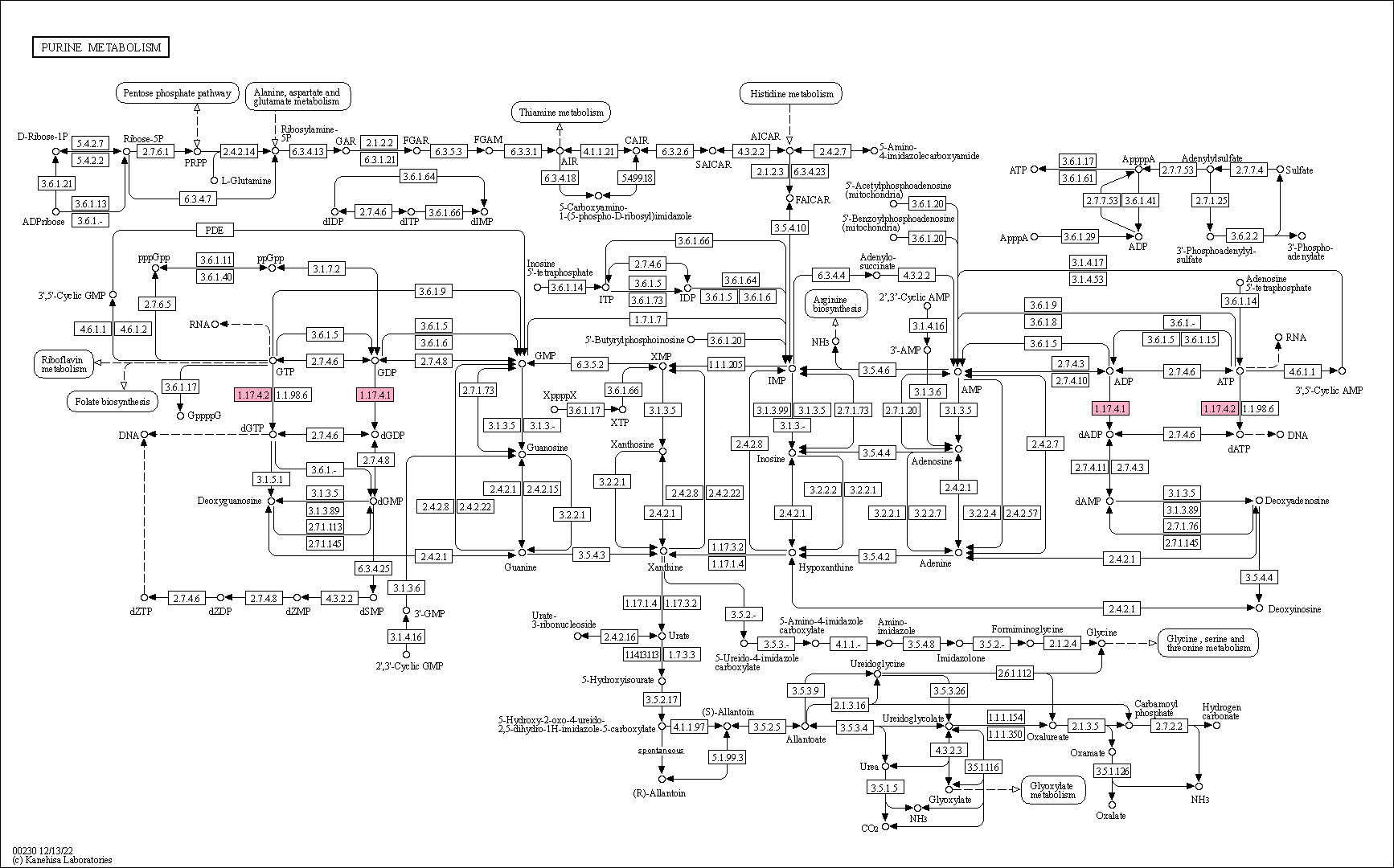
Figure S6. KEGG pathway “Purine metabolism” of sub-category “Nucleotide metabolism” and category “Metabolism” referring to the enriched enzymes in rhizosphere virome of *M. oleifera*, e.g., ribonucleoside-triphosphate reductase (EC 1.17.4.2) and ribonucleoside-diphosphate reductase alpha/beta chain (1.17.4.1). Abundance of these enzymes in the two soil type is shown in Table S15.


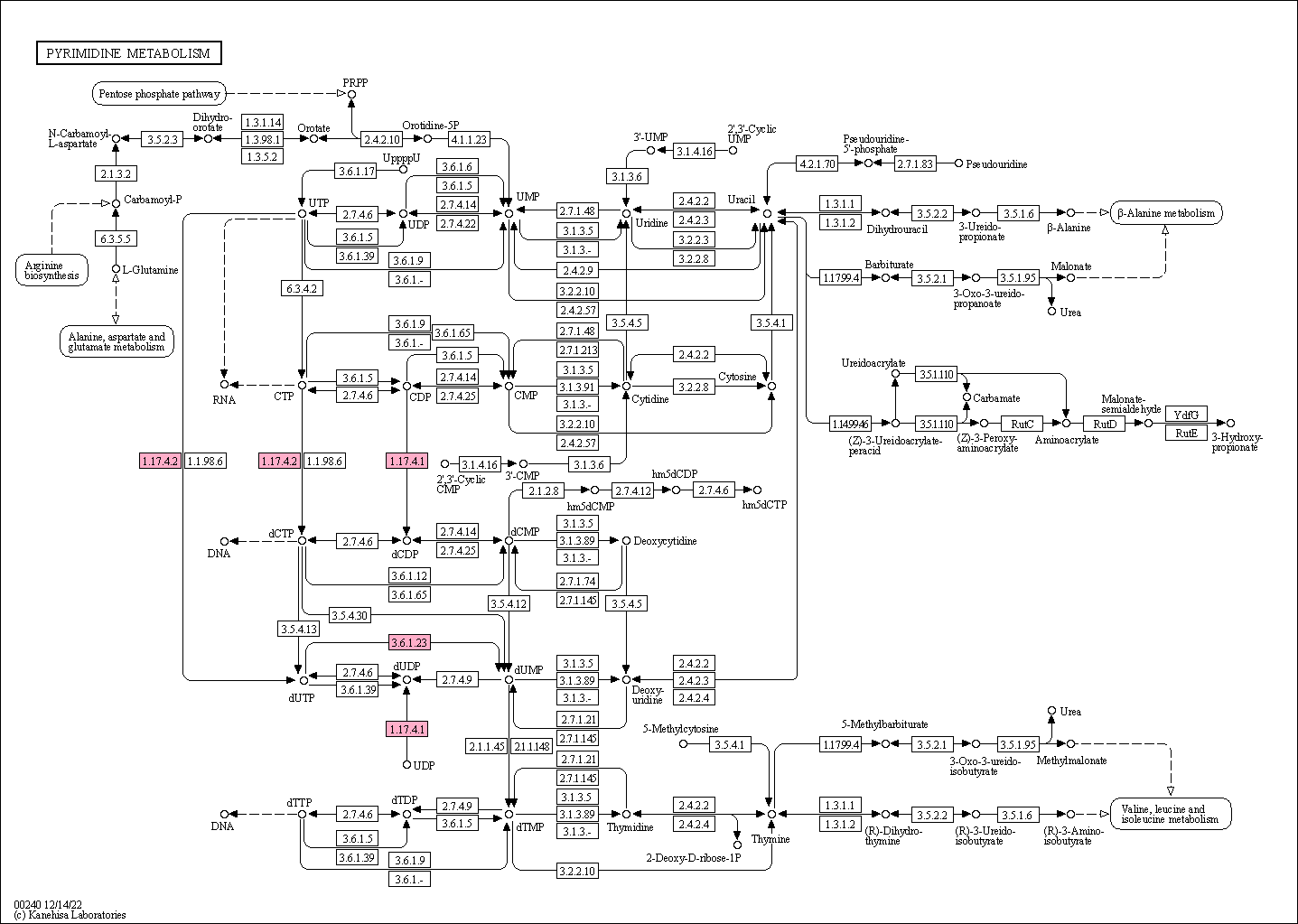
Figure S7. KEGG pathway “Pyrimidine metabolism” of sub-category “Nucleotide metabolism” and category “Metabolism” referring to the three enriched enzymes in rhizosphere virome of *M. oleifera*, e.g., ribonucleoside-triphosphate reductase (EC 1.17.4.2), ribonucleoside-diphosphate reductase alpha/beta chain (1.17.4.1) and dUTP pyrophosphatase (EC 3.6.1.23). Abundance of these enzymes in the two soil type is shown in Table S15.
