# Supplementary material for: Functional annotation of rhizospheric phageome of the wild plant species Moringa oleifera
Source: Front Microbiol. 2023 May 16;14:1166148. doi: 10.3389/fmicb.2023.1166148 (PMC10227523; doi:10.3389/fmicb.2023.1166148)
Supplement: Supplementary file 1 [file Data_Sheet_1.zip › Table S23.docx]

Table S23. Information retrieved from CARD site (<https://card.mcmaster.ca/ontology/>) for the top five highly abundant viral antibiotic resistance genes (ARGs) in rhizosphere of *Moringa oleifera*.

| **CARD gene** | ***soxR*** |
| --- | --- |
| Accession | ARO:3004107 |
| Definition | SoxR is a redox-sensitive transcriptional activator that induces expression of a small regulon that includes the RND efflux pump-encoding operon mexGHI-opmD. SoxR was shown to be activated by pyocyanin. |
| AMR Gene Family | [major facilitator superfamily (MFS) antibiotic efflux pump](https://card.mcmaster.ca/ontology/36003), [ATP-binding cassette (ABC) antibiotic efflux pump](https://card.mcmaster.ca/ontology/36002), [resistance-nodulation-cell division (RND) antibiotic efflux pump](https://card.mcmaster.ca/ontology/36005) |
| Drug Class | [tetracycline antibiotic](https://card.mcmaster.ca/ontology/36189), [fluoroquinolone antibiotic](https://card.mcmaster.ca/ontology/35920), [penam](https://card.mcmaster.ca/ontology/36017), [phenicol antibiotic](https://card.mcmaster.ca/ontology/36526), [cephalosporin](https://card.mcmaster.ca/ontology/35951), [rifamycin antibiotic](https://card.mcmaster.ca/ontology/36296), [disinfecting agents and antiseptics](https://card.mcmaster.ca/ontology/43746), [glycylcycline](https://card.mcmaster.ca/ontology/35960) |
| Resistance Mechanism | [antibiotic efflux](https://card.mcmaster.ca/ontology/36001) |
| Publications | Sakhtah H., et al. The *Pseudomonas aeruginosa* efflux pump MexGHI-OpmD transports a natural phenazine that controls gene expression and biofilm development. *Proc. Natl. Acad. Sci*. U.S.A., **2016**, *113*: E3538- E3547  Palma M., et al. *Pseudomonas aeruginosa* SoxR does not conform to the archetypal paradigm for SoxR-dependent regulation of the bacterial oxidative stress adaptive response. *Infect Immun*, **2005**, *73*: 2958-2966.  Dietrich L.E., et al. The phenazine pyocyanin is a terminal signaling factor in the quorum sensing network of *Pseudomonas aeruginosa*. *Mol. Microbiol*., **2006**, *61*: 1308-1321. |

| **CARD gene** | ***oleC*** |
| --- | --- |
| Accession | ARO:3003748 |
| Definition | oleC is an ABC transporter isolated from *Streptomyces antibioticus* and is involved in oleandomycin secretion. |
| AMR Gene Family | [ATP-binding cassette (ABC) antibiotic efflux pump](https://card.mcmaster.ca/ontology/36002) |
| Drug Class | [macrolide antibiotic](https://card.mcmaster.ca/ontology/35919) |
| Resistance Mechanism | [antibiotic efflux](https://card.mcmaster.ca/ontology/36001) |
| Publications | Rodriguez A.M., et al. *Streptomyces antibioticus* contains at least three oleandomycin-resistance determinants, one of which shows similarity with proteins of the ABC-transporter superfamily. *Mol. Microbiol*., **1993**, *8*: 571-582. |

| **CARD gene** | ***parY* mutant** |
| --- | --- |
| Accession | ARO:3003318 |
| Definition | Intrinsically aminocoumarin-resistant parY variant found in *Streptomyces*, an aminocoumarin-producing genus. |
| AMR Gene Family | [aminocoumarin resistant parY](https://card.mcmaster.ca/ontology/36619) |
| Drug Class | [aminocoumarin antibiotic](https://card.mcmaster.ca/ontology/36242) |
| Resistance Mechanism | [antibiotic target alteration](https://card.mcmaster.ca/ontology/35997) |
| Publications | Schmutz E., et al. 2004. Microbiology 150(PT 3): 641-647. Identification of a topoisomerase IV in actinobacteria: purification and characterization of ParYR and GyrBR from the coumermycin A1 producer *Streptomyces rishiriensis* DSM 40489. |

| **CARD gene** | ***MuxB*** |
| --- | --- |
| Accession | ARO:3004074 |
| Definition | MuxB is one of the two necessary RND components in the *Pseudomonas aeruginosa* efflux pump system MuxABC-OpmB |
| AMR Gene Family | [resistance-nodulation-cell division (RND) antibiotic efflux pump](https://card.mcmaster.ca/ontology/36005) |
| Drug Class | [macrolide antibiotic](https://card.mcmaster.ca/ontology/35919), [monobactam](https://card.mcmaster.ca/ontology/35923), [aminocoumarin antibiotic](https://card.mcmaster.ca/ontology/36242), [tetracycline antibiotic](https://card.mcmaster.ca/ontology/36189) |
| Resistance Mechanism | [antibiotic efflux](https://card.mcmaster.ca/ontology/36001) |
| Publications | Mima, T., et al. Gene cloning and characteristics of the RND-type multidrug efflux pump MuxABC-OpmB possessing two RND components in *Pseudomonas aeruginosa*. *Microbiology*, **2009**, *155*: 3509-3517. |
|  |  |

| **CARD gene** | ***arr-1*** |
| --- | --- |
| Accession | ARO:3002846 |
| Definition | arr-1 is a chromosome-encoded ribosyltransferase found in *Mycolicibacterium smegmatis* |
| AMR Gene Family | [rifampin ADP-ribosyltransferase (Arr)](https://card.mcmaster.ca/ontology/36529) |
| Drug Class | [rifamycin antibiotic](https://card.mcmaster.ca/ontology/36296) |
| Resistance Mechanism | [antibiotic inactivation](https://card.mcmaster.ca/ontology/36000) |
| Publications | Quan, S., et al. Ribosylative inactivation of rifampin by *Mycobacterium smegmatis* is a principal contributor to its low susceptibility to this antibiotic. *Antimicrob Agents Chemother*, **1997**, *41*: 2456-2460. |
